# Synthesis of highly complex phosphorothioate-modified oligonucleotides on microarrays

**DOI:** 10.1038/s41598-026-50718-5

**Published:** 2026-04-28

**Authors:** Igor Ilić, Erika Schaudy, Jory Lietard

**Affiliations:** Institute of Inorganic Chemistry, Faculty of Chemistry, University of Vienna, Josef- Holaubek-Platz 2, Vienna, 1090 Austria

**Keywords:** Biochemistry, Biological techniques, Biotechnology, Chemistry, Drug discovery

## Abstract

The phosphorothioate bond (PS) is one of the most common chemical modifications of DNA and RNA and replaces one of the non-bridging oxygen atoms of the phosphodiester (PO) bond with a sulfur atom. Ubiquitous in oligonucleotide drug design, as it equips most FDA-approved therapeutics, it imparts resistance against nuclease degradation. Racemic PS bonds tend to lower duplex stability, but how and where PS bonds most affect it is difficult to predict. A high-throughput approach such as *in situ* nucleic acid microarray synthesis can potentially help to map out these effects. In this paper, we adapt the sulfurization process to microarray photolithography and carry out the combinatorial synthesis of PO and PS oligos that includes every combination of PO and PS across a set of three different sequences. We were able to map sensitivity to modification and to correlate hybridization signal to melting temperature. Phosphorothioate microarrays should be an ideal platform to accelerate the process of identifying ideal patterns of modifications in a therapeutically relevant context.

**Supplementary Information:**

The online version contains supplementary material available at 10.1038/s41598-026-50718-5.

## Introduction

Oligonucleotide (ODN) drug design necessitates the introduction of chemical modifications in order to overcome the barriers that face unmodified DNA and RNA in a therapeutic context^1–4^. Phosphorothioate (PS) is perhaps the most ubiquitous chemical modification of oligonucleotides as it greatly improves on one of the most critical parameters, namely it increases the resistance of DNA and RNA to nuclease degradation and allows for longer half-lives of nucleic acid therapeutics *in vivo*^5,6^. PS bonds are modifications of the natural phosphodiester linkage between nucleotides where one of the two non-bonding oxygen atoms is substituted for a sulfur atom. This substitution is commonly brought about during ODN synthesis, where the standard oxidation protocol is replaced by a sulfurization step^7–9^. As a result, the phosphorus atom becomes a chiral center, but phosphoramidite-based approaches to synthesizing PS ODNs are not stereoselective and produce PS bonds in racemic mixtures. PS ODNs are effectors of the downregulation of the expression of genes in antisense oligonucleotides and siRNAs^2,4^ and crucially, can still elicit some cleavage by the RNase H enzyme^10^. Phosphorothioates evade degradation by DNases and RNases^11^ but other advantageous properties include increased cellular uptake and intracellular protein affinity^12–14^. On the other hand, association to proteins can cause cellular toxicity^15^ and PS-containing oligonucleotides have a tendency to reduce the affinity of the modified oligonucleotide for its complement^16,17^. A chemical design has to compromise between the desired resistance enhancement^18^ and an undesired, slightly lowered duplex affinity^19^, typically resulting with the so-called gapmer strategy^20^ where the 5ʹ and 3ʹ extremities are PS-modified to protect from exonuclease degradation but the internal section remains unmodified to provide the ideal conditions for RNase H recognition and processing. Better fine-tuning of the physico-chemical properties of PS ODNs is at the heart of synthesizing stereopure PS^21–25^. A more systematic approach to understanding the position, sequence and compounding effect of PS modifications on duplex affinity is difficult to carry out, as the number of possible combinations quickly becomes cumbersome for classical solid-phase synthesis. Recent predictive models of hybridization to PS ODNs as well as other reports^26,27^ have attempted to build a comprehensive dataset of sequence-dependence, though none are looking at a full PO/PS permutational landscape. Nucleic acid microarrays are perfectly poised to explore and achieve permutational completeness due to their synthesis at very high throughput delivering hundreds of thousands of sequence variants^28–34^. In keeping with our mission to broaden the selection of compatible building blocks in nucleic acid photolithography^35–38^, we describe the implementation of a sulfurization procedure to prepare phosphorothioate DNA microarrays which, as far as we are aware, is the first such report in the scientific literature. Our sulfurization is efficient enough to produce PS ODNs that are resistant to nuclease degradation. We then exploit our ability to sulfurize in developing chemical combinatorics where all possible combinations of PO and PS bonds are synthesized and tested at the same time. We chose several sequences of different lengths, yielding up to 131,072 unique PO/PS ODNs that we hybridized to a single complementary strand. The fluorescence intensity allowed us to observe how signal correlates with PS content, with PS-rich ODNs producing lower signal and corresponding with the expected decrease in melting temperature. We are also able to comprehensively map the sensitivity of each phosphorus atom to modification by taking into account every possible PO and PS bond in its immediate vicinity. This work further underlines the usefulness of DNA microarrays as a platform for combinatorics which we expect to be compatible with a large variety of therapeutically relevant modifiers.

## Materials and methods

### Synthesis consumables and reagents

Microarray synthesis was performed using acetonitrile (Sigma-Aldrich L010000), DCI activator (0.25 M in ACN, Sigma-Aldrich L032080), iodine-based oxidizer 0.02 M (THF/H2O/pyridine/iodine 90.54/9.05/0.41/0.43, Sigma-Aldrich L060080), and a 1% solution of imidazole (Sigma-Aldrich 56750) in DMSO (Sigma-Aldrich 34943-M) during UV light exposure. Unreacted hydroxy groups were capped with a mix of fast deprotection Cap A (THF/Tac_2_O 100/5, Sigma-Aldrich L070030) and Cap B (THF/NMI/pyridine 8/1/1, Sigma-Aldrich L050080). Sulfur42 (Sigma-Aldrich M076000) was used at 0.1 M in dry ACN for sulfurization. All nucleoside phosphoramidites used in this study carried a benzoyl-nitrophenylpropyloxycarbonyl (BzNPPOC, Orgentis) photosensitive protecting group at the 5ʹ position. Ethylenediamine (EDA, Sigma-Aldrich 03550) and absolute ethanol (Sigma-Aldrich 1070172511) were used for microarray deprotection. Glass slides are Nexterion D (Schott) and hybridization chambers from Grace Biolabs. Monopodal silane *N*-(3-triethoxysilylpropyl)-4-hydroxybutyramide was from Gelest (SIT8189.5). 5ʹ-Cy3 labelled complementary oligonucleotides were synthesized by Eurogentec.

## Microarray photolithography

*Fabrication generalities.* The process of photolithographic synthesis is based on *in situ* oligonucleotide elongation using phosphoramidite chemistry and the use of terminal photosensitive protecting groups in combination with patterned exposure to UV light *via* a digital micromirror device (DMD). This allows for spatial control of nucleoside coupling and therefore the parallel synthesis of many unique sequences on a single substrate, typically a glass microscope slide. In brief, 365 nm UV light generated by a high-power LED (Nichia NVSU333A) is directed *via* a 0.7’’ DMD (Texas Instruments) onto a pair of pre-silanized microscope glass slides. The chemical synthesis approach consists of consecutive cycles of monomer coupling, capping, oxidation (or sulfurization) and deprotection by exposure to UV light in presence of a solution of 1% imidazole in DMSO. An Expedite 8909 nucleic acid synthesizer (PerSeptive Biosystems) delivers the reagents to pass through a flow cell positioned at the focal distance of the DMD’s image projection. The photolithographic setup allows for the synthesis of up to 786,432 unique oligonucleotides per array.

*Array design.* The list of sequences to be synthesized is fed as a text file onto an in-house built MatLab script (Mathworks) that transforms the list into a single linear sequence that serves as one of the two inputs for the Expedite synthesizer. The script also generates digital masks in the form of bitmap files containing white and black pixels that are understood by the DMD as “ON” or “OFF” exposure, respectively. A digital mask is created for each monomer coupling in the final linear sequence. The script also randomly assigns a position on the array for each sequence. Feature size, or “spot” size, was varied depending on the complexity of the sequence library, with the most complex library using all possible, single-mirror features (~ 14 × 14 μm²) and libraries with the fewest combinations using features of 2 × 2 mirror blocks separated by single-mirror-size of unused space. Microarrays were populated with at least 2 to 3 replicates, and up to ten for the least complex permutation libraries, randomly scattered across the surface.

*Fluidics.* Phosphoramidites, reagents and solvents were loaded onto an Expedite nucleic acid synthesizer, with the auxiliary port charged with the exposure solvent (1% w/v imidazole in DMSO). The fluidics system delivers all reagents to the flow cell, which consists of two glass slides placed on a quartz block, itself encased in a black aluminium support block^39^. The glass slides are separated by a 50 μm-thick PTFE gasket and held in place onto the quartz block with a clamping frame and four screws reaching into the support block with equal torque (5 Nm). Slides and quartz are separated by a 250 μm-thick FFKM gasket. The lower microarray substrate has been customized with two 0.9 mm holes drilled with a CNC router (StepCraft) to align with the two holes on the quartz block. These through-holes allow for the delivery of synthesis reagents onto the flow cell and in between the two array substrates. The flow cell is attached to the synthesizer *via* two 0.4 mm ID PEEK tubing.

## Library synthesis

*Sulfurization.* In general, the decision between backbone sulfurization or oxidation in chemical oligonucleotide synthesis is made after phosphoramidite coupling, exposing the phosphite triester either to oxidizer or to a sulfurizing reagent (0.1 M Sulfur42 in dry ACN). In order to investigate the full backbone permutation library of the tested oligonucleotides, PO and PS variants were synthesized in parallel: each phosphoramidite was delivered to the glass surface twice in a row, with a 30 s oxidation step in the first synthesis cycle, followed by a synthesis cycle with coupling of the same phosphoramidite but with 60 s sulfurization. A different photolithographic mask was used for X + PO and for X + PS at each possible X coupling, allowing for all possible PO/PS variants to be synthesized simultaneously and on the same surface. In addition, a 15 s exposure to capping reagents blocked any unreacted hydroxyl groups in each synthesis cycle. Capping was carried out before oxidation, but after sulfurization^40^.

*Combinatorial PO/PS libraries.* In order to investigate all possible combinations of PO/PS backbone modification in parallel, combinatorial libraries of the three different sequences (13, 15, and 18-nt long) were prepared as permutations of length n-1, resulting in 4,096, 16,384 and 131,072 variants (2^12^, 2^14^ and 2^17^, respectively). A dT_5_ linker (PO) synthesized at the 3ʹ-end ensured equal distance from the glass surface for all variants. Each library was synthesized independently at least three times using 5ʹ-BzNPPOC DNA phosphoramidites^41^.

*Microarray deprotection*. The protecting groups on the nucleobases and phosphodiester bonds were removed in a solution of ethanol and ethylenediamine (1:1 v/v, 2 h at room temperature), followed by a brief wash with RNase-free water. The slides were then dried in a microarray centrifuge and stored in a desiccator until further use.

## Hybridization

Hybridization of microarrays was performed at 37 °C for 2 h with 89 nM Cy3-labeled complement in 1× RNase H reaction buffer (50 mM Tris-HCl, 75 mM KCl, 3 mM MgCl_2_, 10 mM DTT, pH 8.3 @25°C). The hybridization temperature was chosen so as to be below the predicted *T*_m_ of all PO DNA/RNA duplexes at comparable salt concentration (40 °C, 52.8 °C and 60 °C for the 13mer, 15mer and 18mer PO sequences, respectively, and predicted using the OligoAnalyzer tool at idt.com). Hybridization was performed without rotation of the solution in order to homogeneously distribute the fluorescent complement over the array. This precaution mitigates a hybridization artefact whereby rotation of the solution containing the complement leads to noticeably higher signal on the edges of the synthesis area compared to the center. The slides were washed for 10 s in stringent wash buffer (SWB) (100 mM MES, 0.1 M Na^+^, 0.01% Tween 20), final washing buffer (FWB, 0.1× SSC) and dried by centrifugation before scanning.

## Data extraction and analysis

Depending on the feature size, microarrays were either scanned at 5 μm resolution with a GenePix 4100 A or at 2.5 μm resolution with a GenePix 4400 A scanner (Molecular Devices) with a laser excitation set at 532 nm. The scans were aligned to the design layout using NimbleScan 2.1.68 (NimbleGen). Outliers (e.g. features with exceptionally high fluorescence signal due to surface artefacts) were excluded from the raw data set, followed by analysis in Python^42^ and Microsoft Excel. Variants were ranked based on their median fluorescence intensity, then – depending on the size of the dataset – clustered in 25 or 50-sequence bins of decreasing fluorescence signal. The relative PS content (%PS) at each base and each internucleotidic bond within the set of 25 or 50 sequences was calculated for each bin and plotted. To plot the change in %PS per base or linkage as a function of fluorescence signal, a conditional colouring scheme was used to assign a colour to each base/linkage for each bin according to %PS; blue: 0% PS, yellow: 100% PS.

### Degradation assays on PO and PS oligonucleotide microarrays

*Hybridization*. Hybridization tests to assess successful sulfurization were based on the synthesis of the 15mer 5′-TTCGCCGTGTCCCTA on a T_5_ linker at the 3′-end. The complementary probe 5′-TAGGGACACGGCGAA with a 3′-terminal Cy3 modification (Eurogentec) was used for detection in hybridization assays at a concentration of 44.5 nM in MES buffer (100 mM MES, 1 M Na^+^, 20 mM EDTA, 0.01% Tween-20) supplemented with 0.44 mg/mL acetylated BSA (Promega R3961). For hybridization, microarrays were incubated for 45 min at 30 °C with rotation, followed by stringency washes with non-stringent wash buffer (NSWB) (6× SSPE, 0.01% Tween 20) for 2 min, SWB for 1 min and 10 s in FWB. The slides were dried in a microarray centrifuge and scanned at 5 μm resolution. Fluorescent signal intensities were extracted using NimbleScan , followed by data analysis in Microsoft Excel. All on-array reactions were performed in self-adhesive chambers (Grace-Biolabs).

*On-array DNase degradation.* In order to assess the stability of the synthesized oligonucleotides towards enzymatic degradation, one part of the microarray was incubated with 0.02 u/µL DNase I (NEB) for 10 min at 37 °C with rotation in a self-adhesive chamber, while another part on the same glass slide was not exposed to any solution. In order to avoid any contamination to neighbouring parts of the surface, the reaction mixture was carefully removed and the treated part was washed twice by pipetting NSWB in and out of the reaction chamber twice. Finally, the reaction chamber was removed and the slide was washed for 10 s in FWB. Leftover DNA was detected by hybridization (see above) and the susceptibility of DNA towards nuclease degradation was evaluated by comparing hybridization signal intensity between treated and untreated sections of the microarray.

*Off-array DNase degradation and gel analysis*. The 25mer 5′-GTCATCATCATGAACCACCCTGGTC was synthesized on a T_4_ linker and a cleavable dT (thymidine-succinyl hexamide phosphoramidite from ChemGenes, 120 s coupling at 50 mM concentration) using 5′-BzNPPOC phosphoramidites in two separate syntheses using the entire synthesis area, either performing oxidation in every cycle (“25mer_PO”) or sulfurization with Sulfur42 (60 s, 0.1 M solution in ACN) in every 3rd and the final reaction cycle (“25mer_PS”). After deprotection in a mixture of EDA and dry toluene (1:1, both from Sigma) for 2 h at room temperature, the slides were washed with ACN and the DNA was cleaved and collected with 200 µL RNase-free water per slide. After concentration under vacuum at 30 °C, the amount of DNA recovered was quantified with a NanoDrop One (Thermofisher) yielding 78 pmol “25mer_PO” and 99 pmol “25mer_PS”.

19 pmol of each sample were prepared for gel analysis either in 1x DNase I buffer (NEB) as reference, or with additional 0.2 u/µL DNase I (NEB) to study degradation. All samples were incubated for 30 min at 37 °C, mixed 1:1 with 2× RNA loading dye (NEB), heated to 70 °C for 10 min and placed on ice before loading. The 12% PAGE gel was prepared using Rotiphorese Gel Stock solutions (CarlRoth) and supplemented with 7 M urea (Sigma). The gel was pre-run for 15 min at 30 V with TBE 1x running buffer, samples were loaded and after ca. 2 h running at 35 V the gel was stained for 45 min with SYBR gold (Invitrogen S11494) in 1× TBE buffer and scanned with a Fusion FX7 imaging system (Vilber Lourmat).

*T*_*m*_ *measurements.* Measurements were performed on Bioer LineGene 9600 qPCR device, using 96-well plates. Samples were prepared on ice and preparing a master mix for five replicates per sample. The master mix preparation consisted of the following: 5.5 µL test oligo (100 µM), 5.5 µL RNA Complement (100 µM), 5.5 µL EvaGreen (20×), 5.5 µL 10× PBS, 88 µL nuclease-free water. All oligos for *T*_m_ measurements were from Eurogentec, EvaGreen was from Biomol. After the samples were pipetted in the wells (20 µL per well), the 96-well plate was centrifuged. The program settings were as follows: an initial denaturation step at 95 °C for 5 min, ramp down to 20 °C (hold 5 min), followed by stepwise temperature increase from 20 to 100 °C every 0.5 °C for 20 s. Melting temperatures were determined from high-resolution melting curves by numerical differentiation of the fluorescence signal with respect to temperature. For each well, fluorescence values were plotted as a function of temperature, the first derivative dF/dT was calculated, and the negative derivative (-dF/dT) was used to generate derivative melt curves. The *T*_m_ was defined as the temperature at which -dF/dT reached its maximum, corresponding to the point of maximal loss of fluorescence (steepest transition) for that sample.

## Results

**Fig. 1:**
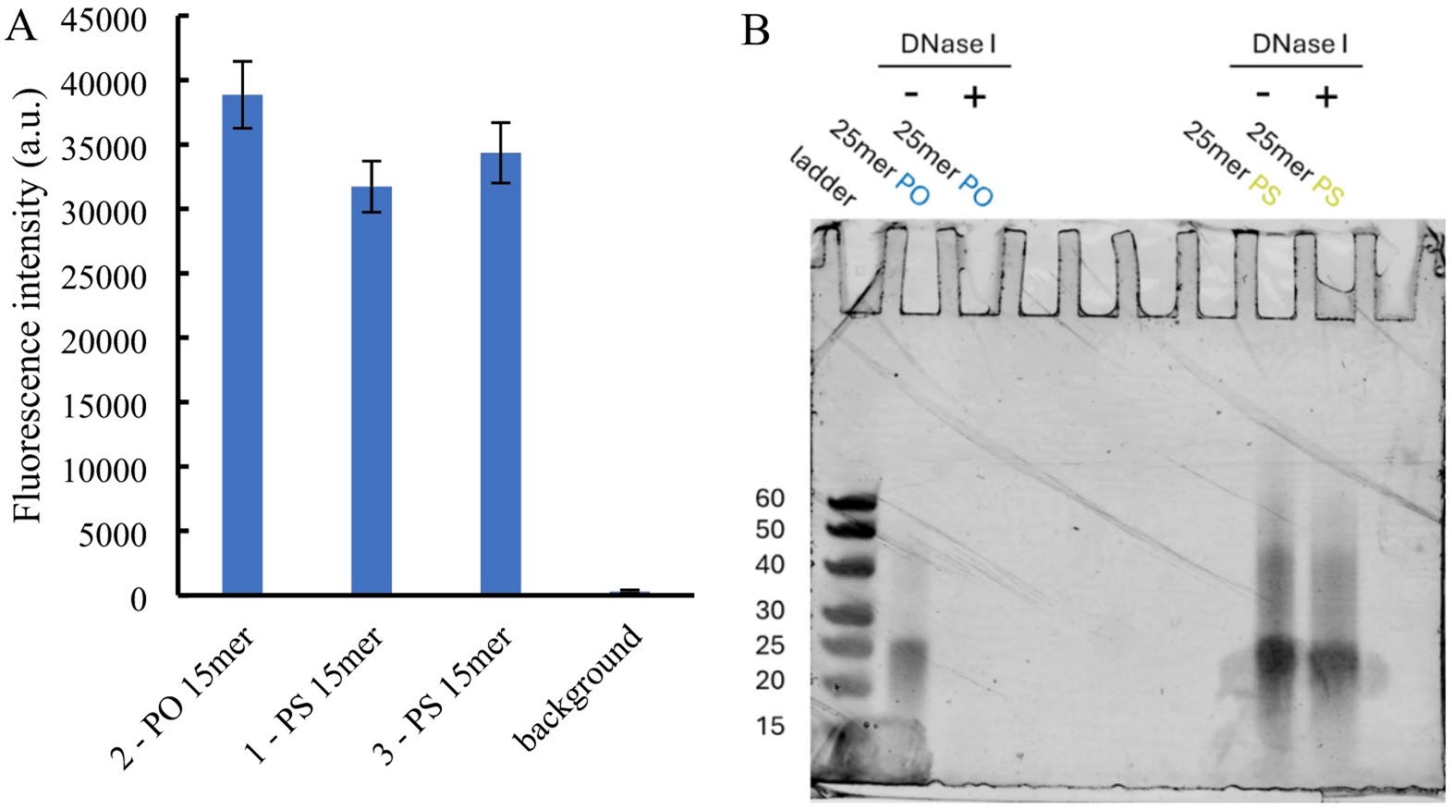
(A) Hybridization assay performed on a microarray populated with a 15mer DNA sequence synthesized in three separate events. The first event is a synthesis with complete sulfurization of all bonds (1 – PS 15mer), the second event is the same sequence synthesized with regular oxidation (2 – PO 15mer) and the final event is a repetition of the first (3 – PS 15mer). Each of the three oligonucleotides are synthesized on separate features. Hybridization is to a Cy3-labeled complementary DNA strand. Error bars are SD. (B) Polyacrylamide gel (12%) analysis of crude oligonucleotide samples cleaved off from the array and treated with DNase I. Synthesis of the 25mer (5ʹ-GTCATCATCATGAACCACCCTGGTCTA) was carried out either with regular iodine-mediated oxidation after each coupling (“25mer PO”) or with Sulfur42-mediated sulfurization (“25mer PS”). After cleavage from the array, an aliquot of 150 ng of DNA was treated with 1 U DNase I (NEB) for 30 min at 37 °C. Aliquots were loaded on the gel (5 µl) with 5 µl of 2× RNA loading dye (NEB). Ladder is a ssDNA ladder (10 to 60 nt).

To perform sulfurization of the phosphite triester during microarray fabrication, we opted for Sulfur42, a 3-phenyl substituted 1,2,4-dithiazole-5-one and close variant of the better known EDITH and ADTT reagents^43,44^, recently introduced and used for the preparation of PS ODNs^45^. We followed the manufacturer’s recommendations and carried out the sulfurization reaction for 1 min with 0.1 M Sulfur42 after each coupling. We should note that this sulfurization process is not stereoselective and produces racemic mixtures of PS bonds, and the ratio of R_p_ vs. S_p_ PS bonds produced with Sulfur42 is unclear. We synthesized a representative 15mer sequence with PS bonds at each internucleotidic linkage and proceeded with deprotection and hybridization with a complementary fluorescent strand. On a PS array, hybridization to a fully-modified PS-15mer yielded high fluorescence intensity indicative of efficient transformation of P(III) to P(V)-sulfur bonds, though somewhat lower than fluorescence intensities recorded for its PO counterpart (Fig. 1A, ~ 39k units for PO-15mer and ~ 32k for PS-15mer).

To account for the small decrease in fluorescence intensity observed for oligonucleotides synthesized earlier in the process of array fabrication^46^, the same PS-15mer was synthesized in a terminal, separate event, now yielding slightly higher hybridization signals at ~ 34k units, but still below those of the PO congener. We then verified the chemical identity of PS oligos grown on microarray by synthesizing a 25mer fully modified with PS bonds, cleaving it from the surface^47^ and retrieving it in solution, then subjecting it to a DNase treatment. The phosphorothioated DNA survived the nuclease treatment while the full-PO equivalent, synthesized using iodine-mediated oxidation, was completely eliminated by DNase I (Fig. 1B). A similar assay performed on array-bound PS and PO oligonucleotides delivered an identical outcome, namely a PS-15mer withstood treatment with the nuclease while its PO congener had been fully degraded (Supplementary Figure S1 and S2). While these enzymatic assays suggest that sulfurization must be very efficient on microarrays, they may not be sensitive enough to detect single PO bonds in otherwise fully modified PS oligos. The desulfurization of PS bonds is a known occurrence and the acidic deblocking step as well as the ammonia-based deprotection have been identified as potential contributors^48,49^, though none of these reagents apply to our photolithographic process except for the use of THF in the capping agents^40^.

**Fig. 2:**
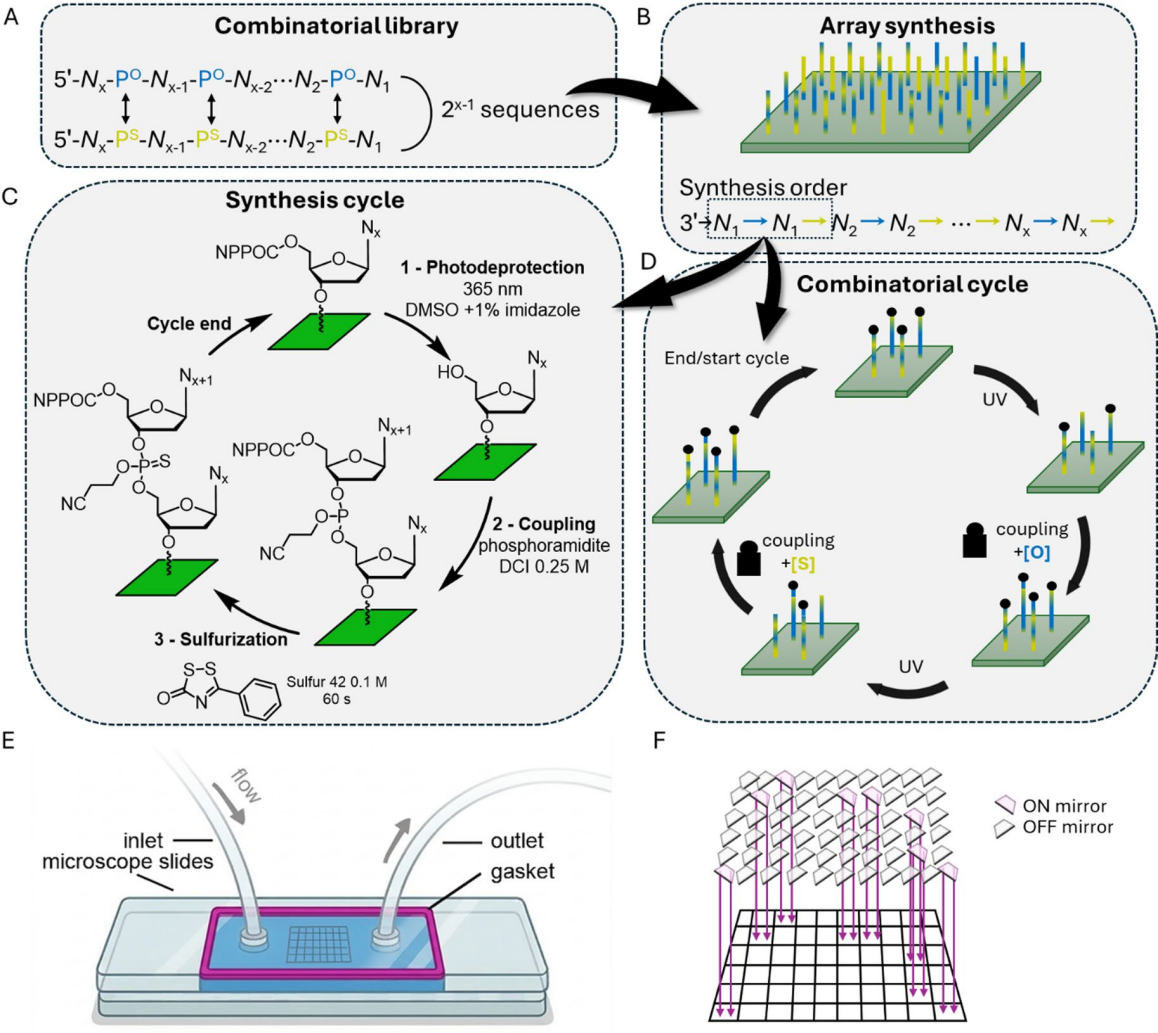
Preparation of combinatorial PO/PS oligonucleotide libraries. (A) For a given sequence of length x and with 2 variables (PO and PS) at each position, the total number of combinations is 2^x-1^. (B) The corresponding library is synthesized in a single run using microarray photolithography, with the synthesis order alternating between oxidation and sulfurization steps after two consecutive phosphoramidite couplings of the same base. (C) Representative sulfurization coupling cycle, with optional capping (step 4) not shown. (D) At the level of the array, the photodeprotection step occurs on half of all growing oligonucleotides before coupling of a given DNA phosphoramidite followed by oxidation, and the remaining half of the sequences couple with the same base followed by sulfurization. (E) Schematic illustration of the flow cell used for microarray photolithography. The assembly consists of two glass microscope slides separated by a thin gasket (purple), creating a microfluidic reaction chamber (in blue). Solvents and reagents flow between the two slides via two holes drilled through one of the two slides. The rectangular grid at the center indicates the synthesis area where all oligonucleotides are grown simultaneously, each section of the grid being independently addressable by UV light. (F) Patterned exposure to 365 nm UV light (in purple) onto the grid-like synthesis area, where each feature of the grid can be independently exposed. To do so, micromirrors of the Digital Micromirror Device are turned either ON or OFF depending on the intended coupling and oxidation type (see Fig. 2D), triggering photodeprotection of the 5ʹ-photosensitive protecting group only at selected positions, and thus allowing for phosphoramidite coupling.

We applied the concept of combinatorial oligonucleotide library to the study of phosphodiester/phosphorothioate (PO/PS) mixtures. We set out to prepare DNA-only libraries where the internucleotidic linkages can be any combination of PO or PS (Fig. 2). This amounts to a 2^*n*−1^ total number of combinations for any *n*-mer. In addition, because the sulfurization is not stereospecific, every PS bond is a mixture of R_p_ and S_p_ isomers, meaning that each sequence synthesized on the array contains a 2^*n*^ number of diasteromers, *n* being the number of PS bonds in the sequence. Each feature on the surface is therefore a 2^n^ family of diastereomeric molecules instead of being a single stereopure species. The implementation of two oxidation protocols in parallel within the process of maskless array synthesis (MAS) is described in Fig. 2C and D. For any nucleotide in the sequence, we proceed with alternating oxidation/sulfurization cycles, i.e. each nucleoside phosphoramidite is coupled twice in separate, consecutive events, the first coupling including an iodine-based oxidation first and the second a sulfurizing oxidation. In practice, before the addition of any new base, half of the array is exposed to UV followed by coupling of amidite X and oxidation (→5ʹ-X_PO_). The other half of the array is then exposed to UV and the corresponding features allowed to couple with amidite X again, only this time followed by sulfurization (→5ʹ-X_PS_). The microfluidic flow cell as well as patterned UV-mediated photodeprotection are shown in Fig. 2E and F.

**Fig. 3:**
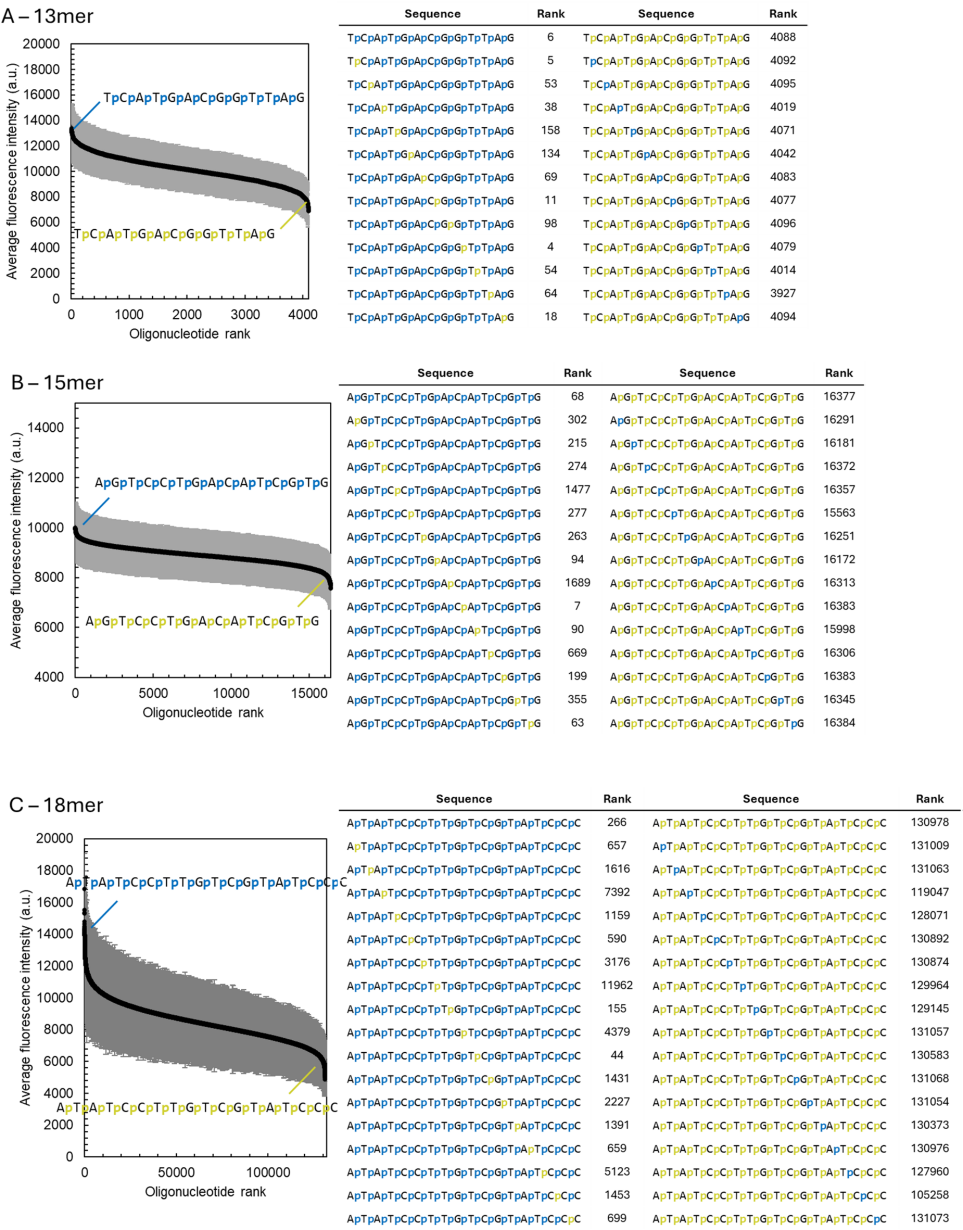
Average fluorescence intensities of hybridization to the PO/PS DNA libraries synthesized as microarrays (*n* = 3 independent syntheses of each). (A) 13mer sequence is 5ʹ-TCATGACGGTTAG, (B) 15mer sequence is 5ʹ-AGTCCTGACATCGTG, (C) 18mer sequence is 5ʹ-ATATCCTTGTCGTATCCC. Intensities are the average of 6 independent measurements. The hybridization signal of the PO-only and PS-only variants of each sequence are localized on each respective curve. The average rank for every oligo with a single PO or a single PS modification is shown in the associated tables on the right. Error bars are sem.

We opted for the synthesis of three different sequences of length 13, 15 and 18 nucleotides, which corresponds to PO/PS microarrays totalling 4096, 16,384 and 131,072 unique oligonucleotides, respectively. Each of these variants can fit in a single microarray with enough space to include multiple replicates of each (11–12 per array for the 15mer, 5–6 per array for the 13mer, and 2–3 per array for the 18mer, see Supplementary Table S1) and each library was synthesized at least three times. The space occupied by each variant on the surface of the array is randomized, and the distribution of technical replicates of each variant is also randomized so as to mitigate the effect of any potential spatial artefacts. The sequence of the 13mer is 5ʹ-TCATGACGGTTAG, that of the 15mer is 5ʹ- AGTCCTGACATCGTG and that of the 18mer 5ʹ-ATATCCTTGTCGTATCCC. After synthesis of the PO/PS DNA libraries of the 13, 15 and 18-nt long sequences, a single hybridization assay with an RNA complement was carried out and the resulting hybridization signals were ranked by intensity (Fig. 3). Indeed, since each library is a single sequence of varying chemical nature, hybridization requires a single complementary ODN to assay each variant simultaneously. Scanning the array surface revealed fluorescent signal intensities of varying strength, suggesting large variations in duplex affinity across all combinations. Lower synthesis quality should also reduce fluorescence signal due to the accumulation of failure sequences, for instance with incomplete phosphoramidite coupling. While the coupling efficiency of regular DNA phosphoramidites in photolithography should be very high (> 99.5%)^50^, not all molecules in a given feature will be error-free and the presence of errors will decrease hybridization affinity. However, given that every variant in the PO/PS libraries is, at its core, the same sequence with the same number and the same types of amidite couplings at every step, the percentage of failure sequences is expected to be uniform across all chemical variants, reducing the overall brightness of the array scan in a variant-independent manner. The range of fluorescence is normally distributed in all three cases, within the linear range of fluorescence detection on conventional microarray scanner and far below saturation levels^51^. The range spans several thousands of units between the brightest and the dimmest spots (~ 7k range for the 13mer, ~ 2.5k for the 15mer and ~9k for the 18mer).

In all three cases, the unmodified PO-only variant emerges as some of the highest in terms of hybridization signal intensity (#6 out of 4096, #68 out of 16,384 and #266 out of 131,072 for the 13, 15 and 18mer, respectively) while the PS-only variant hybridizes with some of the lowest signals (#4088, #16,377 and #130,978). Low signal is indicative of lower duplex affinity, which is line with the well-known decrease of stability of PS ODN, estimated at ~-0.5 °C per PS bond^16,19,26,52^. Differences in the range of fluorescence could stem from several technical and sequence-related factors contributing to varying degrees. First, the set hybridization temperature (37 °C) will likely have a more pronounced effect on duplex stability for the shorter 13mer compared to the longer 15 and 18mers, particularly in the case of the less stable PS-rich variants, which should produce an increased range of hybridization signals. Second, for the more stable oligomers at the same assay temperature, the wider fluorescence range for the 18mer could be due to a larger range of duplex stabilities between the unmodified and fully modified PS-18mer. Finally, the lower number of technical replicates for the 18mer library may result in lower data accuracy and extended tails in the distribution of signals.

Variants of interest can be located individually (Fig. 3A). In the 13mer, ODNs carrying a single PS mod are all located below the full PO ODN, suggesting a slight negative effect on *T*_m_, but a PS bond at the 5ʹ extremity (5ʹ-T_PS_C) and an internal PS at the G_9_/T_10_ step rank close to the full PO ODN. For full PS backbones, variants with a single PO at the 5ʹ or 3ʹ extremity rank close to the full PS-13mer, suggesting little improvement in duplex affinity. Most other single PO variants rank above that of the full PS-13mer, indicating increasing stability, except for the G_8_/G_9_ step. In the case of the 15mer (Fig. 3B), single PS bond ODNs rank lower than the unmodified 15mer except for a C_9_/A_10_ step. Single PO bonds within a fully modified PS backbone globally rank higher than the PS-only 15mer, once again indicating a slight increase in duplex affinity but, interestingly, a T_3_/C_4_, as well as a C_12_/G_13_ and a T_14_/G_15_ towards the 3ʹ end are at the level of the PS-only 15mer, suggesting no noticeable increase in stability when leaving those single phosphorus atoms unmodified. In the 18mer (Fig. 3C), of all 17 possible single PS modifications, only those at T_PS_G and T_PS_C, centrally located, rank close to the native PO-only oligo, while all others are found much further down the rank list. At the bottom end of the fluorescence curve where PS-rich oligos are located, single PO variants rank close or slightly higher than the PS-only 18mer, but notable exceptions are an A_3_/T_4_ and a C_16_/C_17_ steps, found much higher on the list and suggesting increased stability. It is important to note that the G_PS_T step found in the 13mer with little impact on fluorescence relative to the unmodified PO-13mer is not seen in the other two sequences. There, G_PS_T steps decrease fluorescence signal relative to the unmodified variant. This would indicate that the effect of phosphorothioate on duplex stability goes beyond the identity of the neighbouring bases, and broader sequence context as well as length and position of the modification need to be taken into account. It should, however, be borne in mind that any observation relies on the interpretation of the effect of diastereomeric PS mixtures on duplex affinity, despite the well-established fact that R_p_ and S_p_ isomers differently affect duplex affinity. The positional sensitivity may, in addition, simply reflect variations in R_p_/S_p_ ratios at specific positions rather than a diastereomeric average. It is unclear whether the diastereoselectivity of Sulfur42-mediated sulfurization depends on position and sequence context or changes as the synthesis progresses.

**Fig. 4:**
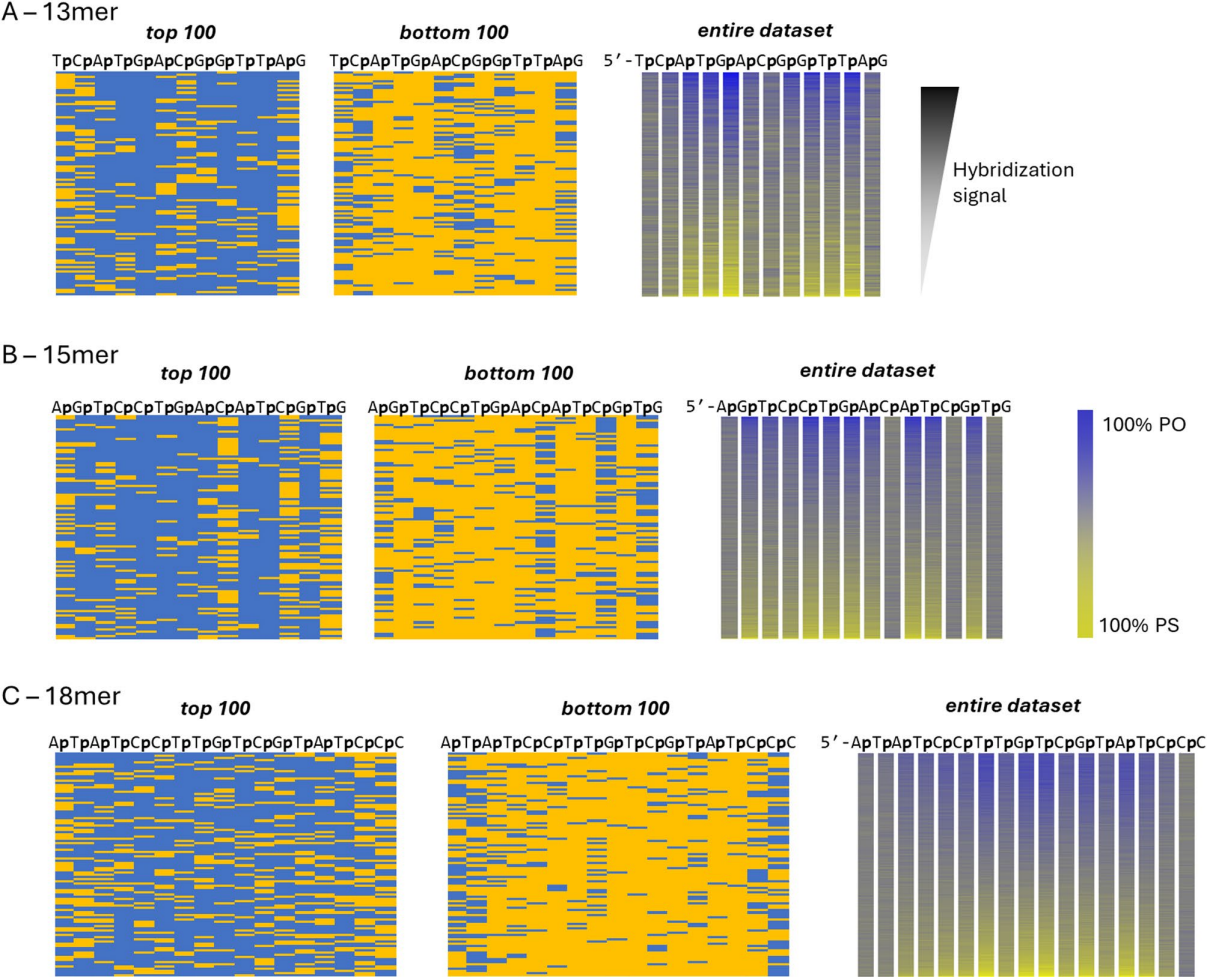
PO/PS distribution per linkage and as a function of hybridization signal for the (A) 13mer, (B) 15mer and (C) 18mer. Left and middle plots represent the top and bottom 100 sequences, each of the 100 sequences being given a colour corresponding to either PO (blue) or PS (yellow) chemistry found at every phosphorus. The right-most plot is the binned distribution of fluorescence (bin size = 50 sequences for 15mer and 18mer, 25 sequences for 13mer) arranged by internucleotidic linkage. For each bin at each linkage, a colour code was assigned corresponding to the relative frequency of PO or PS at that position. Bins are arranged from high to low fluorescence, top to bottom.

To examine the preference and sensitivity per linkage, for all linkages and at the same time and track it along the fluorescence curve, the fluorescence data was arranged and cut into bins of 50 sequences for the 15 and 18mer, and 25 sequences for the 13mer, and the bins themselves were arranged from high to low fluorescence, for every linkage (Fig. 4 and Supplementary Figure S3, S4 and S5).

For each bin and for each linkage, we assign a color to that bin that corresponds to the relative frequency of PO or PS found in the set of oligos. A strong blue color corresponds to close to 100% PO chemistry in the oligos contained within a given bin, and a strong yellow color corresponds to 100% PS chemistry, and a gradient between these two extremes representing every other possible PO/PS ratio (Fig. 4B). In so doing, we can track the prevalence of PO or PS chemistry for each linkage along the hybridization curve. Looking at the distribution of PO and PS bonds at each internucleotidic linkage and across the entire range of hybridization signals, some patterns emerge. First, the top range of fluorescence intensities is always populated with PO-rich variants for all three sequences and, conversely, the lowest range is populated with PS-rich oligonucleotides, indicating lower affinity. This is particularly noticeable in the list of top 100 and bottom 100 variants for each sequence where any blue marker indicates a PO bond and any yellow marker a PS bond. Second, for all three sequences, both the 5ʹ and the 3ʹ ends appear insensitive to the nature of the non-bonding atom at the phosphorus, and for the 18mer this is true for the first two linkages from both extremities. This is visible in the top and bottom 100 list, where the opposite color populates the 5ʹ and 3ʹ end in close to 50% of the variants. In the bin plots, the insensitivity of the extremities is noticeable with the lack of clear color transition from top to bottom, showing instead a constant blend of blue and yellow. It is perhaps not surprising to observe low sensitivity of the extremities to chemical changes, as it is well known that 5ʹ and 3ʹ ends better tolerate modifications than central regions of the duplex. However, in surface-bound assays such as hybridization on microarrays, the 3ʹ-end is covalently tethered to the surface via a nucleotidic T-linker whereas the 5ʹ-end is in its native 5ʹ-OH form and in direct contact with the aqueous environment. Whether this attachment asymmetry exacerbates, reduces or has any effect on the sensitivity of the 3ʹ-end relative to the 5ʹ-end does not seem immediately apparent, but cannot be ruled out. We find that the sensitivity of oxygen replacement by sulfur increases unfavourably for sulfur towards the middle of the oligonucleotide with, as expected, a predominance of PO chemistry for internal bonds in the high fluorescence range. Across all sequences, no single internal linkage strongly prefers PS to increase hybridization signal relative to PO. Nonetheless, a select number of very central bonds are only moderately sensitive to sulfurization: between A_8_/C_9_ and between C_9_/G_10_ in the 13mer (60% PO content in the top sequences down to ~ 35% PO content in the bottom range), C_9_/A_10_ in the 15mer (45% PO→55% PO), and T_8_/G_9_ and C_11_/G_12_ in the 18mer (60% PO→20% PO). The 5ʹ-C_PS_G step in the 13mer, the 5ʹ-C_PS_A step in the 15mer and the 5ʹ-T_PS_G step in the 18mer were ranked close to the full PO in the single modification list (Fig. 3). For all other internal bonds, the PO content decreases with fluorescence intensity from 65 to 80% down to < 10%. We find that our oligomers have a decent proportion of PS in the top fluorescence of C_PS_G steps and can accommodate T_PS_A steps better than in A_PS_T steps, which is in line with the reported lower stability of phosphorothioate A_PS_T and G_PS_C relative to T_PS_A and C_PS_G bonds^27,53^. The presence of C_PS_A and A_PS_C in the top range of fluorescence is also perhaps not unexpected, as the free energy contribution of C_PS_A and A_PS_C bonds was predicted to sit at -0.82 and  -0.88 kcal/mol, close to the PO equivalent (-0.95 and  -0.94 kcal/mol)^26^. Whether these motifs and this positional sensitivity would be detectable in other sequences is speculative, and carrying out such broad generalizations would in any case require scanning an extensive and comprehensive sequence space. And while photolithographic nucleic acid synthesis can produce hundreds of thousands of unique sequences in parallel, exploring both sequence and chemical space simultaneously on a single array is beyond the capacity of maskless array synthesis. We also tried to increase probe accessibility by increasing salt concentration in the hybridization solution, so as to counter the potentially dense network of negatively charged molecules on the array. But we find that an increased cation concentration does not noticeably affect the positional sensitivity to PO or PS chemistry, as indicated by similar patterns in the 13mer with 1 M Na^+^ instead of 75 mM (Supplementary Figure S6).

**Fig. 5:**
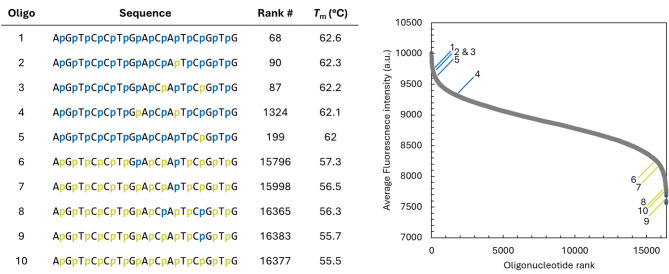
(left) Recorded *T*_m_ of oligonucleotides 1–10 of the 15mer sequence with varying PO (blue) and PS (yellow) content, with their corresponding average fluorescence ranking on the PO/PS microarray. (right) approximate location of each of the ten oligos on the fluorescence distribution curve.

We measured the *T*_m_ of a few PO/PS oligonucleotide candidates of the 15mer sequence with its RNA complement using high resolution melting experiments (Supplementary Figure S7). We selected positions that appeared populated with either PS or PO chemistries in the tail ends of the fluorescence curves. We found that the measured *T*_m_s broadly map with rankings (Fig. 5). PO-rich sequences bind to the RNA complement with *T*_m_s of ~ 62 °C while the duplexes with PS-rich counterparts have inferior *T*_m_s, centered around ~ 56 °C. This corresponds to variations in fluorescence, with PS-rich oligonucleotides consistently yielding lower hybridization signals. Of the PO-rich sequences, ODN 5 with a PS bond between C_12_ and G_13_ fares slightly worse (62 °C) than ODN 3 with PS bonds between C_12_/G_13_ and C_9_/A_10_ (62.2 °C), which corresponds to fluorescence ranking. This result indicates that the incorporation of multiple PS bonds does not always lead to stepwise decrease in duplex affinity, which our microarray approach was able to predict for this particular sequence. For PS-rich sequences, we find that ODN 6 with PO bonds between G_7_/A_8_ and A_10_/T_11_ has a higher *T*_m_ (57.3 °C) than ODN 8 with PO bonds between C_9_/A_10_ and C_12_/G_13_ (56.3 °C), strongly correlating with fluorescence and sensitivity of these positions to modifications (Fig. 5B). The PO-rich sequences 1 to 5 have a maximum *T*_m_ range of ~ 0.6 °C and that of the PS-rich sequences 6 to 10 is three times as high, at ~ 1.8 °C. The range of fluorescence signal for ODNs 1 to 5 is ~ 130 units and the range of fluorescence signal for ODNs 6 to 10 is a little over three times as great, at ~ 480 units, which suggests that for the 15mer hybridization data, variations of fluorescence signal correlate with changes in *T*_m_, at least for the selected fluorescence region. *T*_m_-fluorescence correlation appears to be valid for this subset of 15-nt long oligonucleotides, but whether this is true for all other oligonucleotide sequences tested herein, as well as broadly generalizable, remains to be demonstrated.

## Conclusion

In this paper, we adapt standard procedures for sulfurization in oligonucleotide solid-phase synthesis for the preparation of racemic phosphorothioate DNA microarrays. The sulfurization process is efficient, as evidenced by the grown PS DNA sequences able to resist nuclease-mediated degradation, on or off-array, though these control assays cannot guarantee that a complete, 100% sulfurization rate has consistently been achieved at each and every step. We use our ability to sulfurize during array fabrication to explore the combinatorial synthesis of oligonucleotide libraries containing all possible PO and PS modifications, reaching up to hundreds of thousands of unique PO/PS variants that could also be synthesized and assayed simultaneously. We performed a standard hybridization assay using a complementary strand to understand how patterns of chemical modifications affect fluorescence signal. We find that fluorescence hybridization signal is able to portray changes in duplex affinity on microarrays, which had been demonstrated in the case of changes in nucleotide content^54^, but in the case of fixed sequences with varying chemical composition it had, to our knowledge, not previously been reported on. Further work will attempt to shed light on the thermodynamic elements of surface hybridization with chemically modified oligonucleotides by varying the concentration of the fluorescent complement. Some limitations of the approach should be outlined: because the sulfurization is not stereoselective, PS oligonucleotides on the array are diastereomeric mixtures and the hybridization data reflects the behaviour of racemic PS compounds rather than that of chirally-defined sulfurized species. Second, due to the small subset of sequences tested in this study, considerations on the sequence and positional sensitivity of phosphorothioate bonds should be seen as valid for this subset only and may not be generalizable beyond that. Nevertheless, this paper further illustrates the potential of nucleic acid microarray synthesis *in situ* for the massively parallel preparation of oligo variants. Future work will showcase how additional, therapeutically relevant chemical modifications are also compatible with microarray photolithography.

## Supplementary Information

Below is the link to the electronic supplementary material.

Supplementary Material 1

## Data Availability

The data accompanying this article is available as supplementary material. Fluorescence scans and raw data files can be forwarded upon reasonable request to the corresponding author.

## References

[1] Quemener, A. M. et al. D. The powerful world of antisense oligonucleotides: From bench to bedside. *Wiley interdisciplinary reviews RNA* **11** (5), e1594. (2020). 10.1002/wrna.1594 PMC9285911 32233021

[2] Roberts, T. C., Langer, R. & Wood, M. J. A. Advances in oligonucleotide drug delivery. *Nat. Rev. Drug Discov.* **19** (10), 673–694 (2020). 32782413 10.1038/s41573-020-0075-7 PMC7419031

[3] Sun, X., Setrerrahmane, S., Li, C., Hu, J. & Xu, H. Nucleic acid drugs: recent progress and future perspectives. *Signal. Transduct. Target. Ther.* **9** (1), 316 (2024). 39609384 10.1038/s41392-024-02035-4 PMC11604671

[4] Egli, M. & Manoharan, M. Chemistry, structure and function of approved oligonucleotide therapeutics. *Nucleic Acids Res.* **51** (6), 2529–2573 (2023). 36881759 10.1093/nar/gkad067 PMC10085713

[5] Eckstein, F. Phosphorothioates, essential components of therapeutic oligonucleotides. *Nucleic Acid Ther.* **24** (6), 374–387 (2014). 25353652 10.1089/nat.2014.0506

[6] Clave, G., Reverte, M., Vasseur, J. J. & Smietana, M. Modified internucleoside linkages for nuclease-resistant oligonucleotides. *RSC Chem. Biol.* **2** (1), 94–150 (2021). 34458777 10.1039/d0cb00136h PMC8341215

[7] Scotson, J. L., Andrews, B. I., Laws, A. P. & Page, M. I. Phosphorothioate anti-sense oligonucleotides: the kinetics and mechanism of the generation of the sulfurising agent from phenylacetyl disulfide (PADS). *Org. Biomol. Chem.* **14** (35), 8301–8308 (2016). 27531007 10.1039/c6ob01531j

[8] Krotz, A. H. et al. Phosphorothioate Oligonucleotides with Low Phosphate Diester Content: Greater than 99.9% Sulfurization Efficiency with Aged Solutions of Phenylacetyl Disulfide (PADS). *Org. Process. Res. Dev.* **8** (6), 852–858 (2004).

[9] Tang, J. Y., Han, Y., Tang, J. X. & Zhang, Z. Large-Scale Synthesis of Oligonucleotide Phosphorothioates Using 3-Amino-1,2,4-dithiazole-5-thione as an Efficient Sulfur-Transfer Reagent. *Org. Process. Res. Dev.* **4** (3), 194–198 (2000).

[10] Furdon, P. J., Dominski, Z., Kole, R. & RNase H cleavage of RNA hybridized to oligonucleotides containing methylphosphonate, phosphorothioate and phosphodiester bonds. *Nucleic Acids Res.* **17** (22), 9193–9204 (1989). 2555787 10.1093/nar/17.22.9193 PMC335124

[11] Spitzer, S. & Eckstein, F. Inhibition of deoxyribonucleases by phosphorothioate groups in oligodeoxyribonucleotides. *Nucleic Acids Res.* **16** (24), 11691–11704 (1988). 2850541 10.1093/nar/16.24.11691 PMC339104

[12] Bijsterbosch, M. K. et al. In vivo fate of phosphorothioate antisense oligodeoxynucleotides: predominant uptake by scavenger receptors on endothelial liver cells. *Nucleic Acids Res.* **25** (16), 3290–3296 (1997). 9241243 10.1093/nar/25.16.3290 PMC146893

[13] Miller, C. M. et al. Stabilin-1 and Stabilin-2 are specific receptors for the cellular internalization of phosphorothioate-modified antisense oligonucleotides (ASOs) in the liver. *Nucleic Acids Res.* **44** (6), 2782–2794 (2016). 26908652 10.1093/nar/gkw112 PMC4824115

[14] Liang, X. H., Sun, H., Shen, W. & Crooke, S. T. Identification and characterization of intracellular proteins that bind oligonucleotides with phosphorothioate linkages. *Nucleic Acids Res.* **43** (5), 2927–2945 (2015). 25712094 10.1093/nar/gkv143 PMC4357732

[15] Crooke, S. T., Seth, P. P., Vickers, T. A. & Liang, X. H. The Interaction of Phosphorothioate-Containing RNA Targeted Drugs with Proteins Is a Critical Determinant of the Therapeutic Effects of These Agents. *J. Am. Chem. Soc.* **142** (35), 14754–14771 (2020). 32786803 10.1021/jacs.0c04928

[16] Stein, C. A., Subasinghe, C., Shinozuka, K. & Cohen, J. S. Physicochemical properties of phosphorothioate oligodeoxynucleotides. *Nucleic Acids Res.* **16** (8), 3209–3221 (1988). 2836790 10.1093/nar/16.8.3209 PMC336489

[17] Clark, C. L., Cecil, P. K., Singh, D. & Gray, D. M. CD, absorption and thermodynamic analysis of repeating dinucleotide DNA, RNA and hybrid duplexes [d/r(AC)]12.[d/r(GT/U)]12 and the influence of phosphorothioate substitution. *Nucleic Acids Res.* **25** (20), 4098–4105 (1997). 9321664 10.1093/nar/25.20.4098 PMC147004

[18] Ciafre, S. A. et al. Stability and functional effectiveness of phosphorothioate modified duplex DNA and synthetic ‘mini-genes’. *Nucleic Acids Res.* **23** (20), 4134–4142 (1995). 7479077 10.1093/nar/23.20.4134 PMC307355

[19] Jaroszewski, J. W., Clausen, V., Cohen, J. S. & Dahl, O. NMR investigations of duplex stability of phosphorothioate and phosphorodithioate DNA analogues modified in both strands. *Nucleic Acids Res.* **24** (5), 829–834 (1996). 8600448 10.1093/nar/24.5.829 PMC145729

[20] Monia, B. P. et al. Evaluation of 2‘-modified oligonucleotides containing 2‘-deoxy gaps as antisense inhibitors of gene expression. *J. Biol. Chem.* **268** (19), 14514–14522 (1993). 8390996

[21] Stec, W. J. et al. Deoxyribonucleoside 3 ‘-O-(2-thio- and 2-oxo-spiro-4,4-pentamethylene-1,3,2-oxathiaphospholane)s: Monomers for stereocontrolled synthesis of oligo(deoxyribonucleoside phosphorothioate)s and chimeric PS/PO oligonucleotides. *J. Am. Chem. Soc.* **120** (29), 7156–7167 (1998).

[22] Iwamoto, N. et al. Control of phosphorothioate stereochemistry substantially increases the efficacy of antisense oligonucleotides. *Nat. Biotechnol.* **35** (9), 845–851 (2017). 28829437 10.1038/nbt.3948

[23] Knouse, K. W. et al. Unlocking P(V): Reagents for chiral phosphorothioate synthesis. *Science* **361** (6408), 1234–1238 (2018). 30072577 10.1126/science.aau3369 PMC6349427

[24] Ostergaard, M. E. et al. Understanding the effect of controlling phosphorothioate chirality in the DNA gap on the potency and safety of gapmer antisense oligonucleotides. *Nucleic Acids Res.* **48** (4), 1691–1700 (2020). 31980820 10.1093/nar/gkaa031 PMC7038945

[25] Rosenqvist, P. et al. Stereo-Controlled Liquid Phase Synthesis of Phosphorothioate Oligonucleotides on a Soluble Support. *J. Org. Chem.* **88** (14), 10156–10163 (2023). 37428953 10.1021/acs.joc.3c01006 PMC10367069

[26] Wang, S. S., Xiong, E., Bhadra, S. & Ellington, A. D. Developing predictive hybridization models for phosphorothioate oligonucleotides using high-resolution melting. *PLoS One* **17** (5), e0268575. (2022). 10.1371/journal.pone.0268575 PMC9116672 35584176

[27] Hartmann, B., Bertrand, H. & Fermandjian, S. Sequence effects on energetic and structural properties of phosphorothioate DNA: a molecular modelling study. *Nucleic Acids Res.* **27** (16), 3342–3347 (1999). 10454642 10.1093/nar/27.16.3342 PMC148568

[28] Behr, J. et al. An open-source advanced maskless synthesizer for light-directed chemical synthesis of large nucleic acid libraries and microarrays. *ChemRxiv* (2024).

[29] Srivannavit, O. et al. Design and fabrication of microwell array chips for a solution-based, photogenerated acid-catalyzed parallel oligonuclotide DNA synthesis. *Sens. Actuators A: Phys.* **116** (1), 150–160 (2004).

[30] LeProust, E. M. et al. Synthesis of high-quality libraries of long (150mer) oligonucleotides by a novel depurination controlled process. *Nucleic Acids Res.* **38** (8), 2522–2540 (2010). 20308161 10.1093/nar/gkq163 PMC2860131

[31] Singh-Gasson, S. et al. Maskless fabrication of light-directed oligonucleotide microarrays using a digital micromirror array. *Nat. Biotechnol.* **17** (10), 974–978. 10.1038/13664 (1999). 10504697 10.1038/13664

[32] Ghindilis, A. L. et al. CombiMatrix oligonucleotide arrays: genotyping and gene expression assays employing electrochemical detection. *Biosens. Bioelectron.* **22** (9–10), 1853–1860 (2007). 16891109 10.1016/j.bios.2006.06.024

[33] Lietard, J., Ameur, D., Damha, M. & Somoza, M. M. High-density RNA microarrays synthesized in situ by photolithography. *Angew Chem. Int. Ed.* **57** (46), 15257–15261 (2018). 10.1002/anie.201806895 PMC6237118 30187993

[34] Kekic, T. et al. Accelerated, high-quality photolithographic synthesis of RNA microarrays in situ. *Sci. Adv.* **10** (31), eado6762 (2024). 39083603 10.1126/sciadv.ado6762 PMC11290486

[35] Lietard, J. et al. Mapping the affinity landscape of Thrombin-binding aptamers on 2’F-ANA/DNA chimeric G-Quadruplex microarrays. *Nucleic Acids Res.* **45** (4), 1619–1632 (2017). 28100695 10.1093/nar/gkw1357 PMC5389548

[36] Holz, K., Schaudy, E., Lietard, J. & Somoza, M. M. Multi-level patterning nucleic acid photolithography. *Nat. Commun.* **10** (1), 3805 (2019). 31444344 10.1038/s41467-019-11670-3 PMC6707258

[37] Schaudy, E., Somoza, M. M. & Lietard, J. l-DNA Duplex Formation as a Bioorthogonal Information Channel in Nucleic Acid-Based Surface Patterning. *Chem. Eur. J.* **26** (63), 14310–14314 (2020). 32515523 10.1002/chem.202001871 PMC7702103

[38] Schaudy, E., Lietard, J. & Somoza, M. M. Sequence Preference and Initiator Promiscuity for De Novo DNA Synthesis by Terminal Deoxynucleotidyl Transferase. *ACS Synth. Biol.* **10** (7), 1750–1760 (2021). 34156829 10.1021/acssynbio.1c00142 PMC8291772

[39] Sack, M., Kretschy, N., Rohm, B., Somoza, V. & Somoza, M. M. Simultaneous Light-Directed Synthesis of Mirror-Image Microarrays in a Photochemical Reaction Cell with Flare Suppression. *Anal. Chem.* **85** (18), 8513–8517 (2013). 23968455 10.1021/ac4024318 PMC3776564

[40] Ren, Q., Osawa, T., Tatsuno, M. & Obika, S. THF peroxide as a factor in generating desulphurised products from the solid-phase synthesis of phosphorothioate-modified oligonucleotides. *RSC Adv.* **14** (30), 21590–21596 (2024). 38979452 10.1039/d4ra03592e PMC11229082

[41] Kretschy, N., Holik, A. K., Somoza, V., Stengele, K. P. & Somoza, M. M. Next-Generation o-Nitrobenzyl Photolabile Groups for Light-Directed Chemistry and Microarray Synthesis. *Angew Chem. Int. Ed.* **54** (29), 8555–8559 (2015). 10.1002/anie.201502125 PMC4531821 26036777

[42] Kekic, T. & Lietard, J. A. Canvas of Spatially Arranged DNA Strands that Can Produce 24-bit Color Depth. *J. Am. Chem. Soc.* **145** (41), 22293–22297 (2023). 37787949 10.1021/jacs.3c06500 PMC10591465

[43] Ponomarov, O., Laws, A. P. & Hanusek, J. 1,2,4-Dithiazole-5-ones and 5-thiones as efficient sulfurizing agents of phosphorus(III) compounds–a kinetic comparative study. *Org. Biomol. Chem.* **10** (44), 8868–8876 (2012). 23052107 10.1039/c2ob26460a

[44] Volk, D. E. & Lokesh, G. L. R. Development of Phosphorothioate DNA and DNA Thioaptamers. *Biomedicines* **5** (3). (2017). 10.3390/biomedicines5030041 PMC5618299 28703779

[45] Chen, J., Chen, M. & Zhu, T. F. Translating protein enzymes without aminoacyl-tRNA synthetases. *Chem* **7** (3), 786–798 (2021).

[46] Sack, M. et al. Express photolithographic DNA microarray synthesis with optimized chemistry and high-efficiency photolabile groups. *J. Nanobiotechnol.* **14**, 1–13 (2016). 10.1186/s12951-016-0166-0 PMC4776362 26936369

[47] Lietard, J. et al. Base-cleavable microarrays for the characterization of DNA and RNA oligonucleotides synthesized in situ by photolithography. *Chem. Commun.* **50** (85), 12903–12906 (2014). 10.1039/c4cc05771f PMC4183992 25213224

[48] Cieslak, J. et al. L. 31P NMR study of the desulfurization of oligonucleoside phosphorothioates effected by aged trichloroacetic acid solutions. *J. Org. Chem.* **70** (8), 3303–3306 (2005). 15823001 10.1021/jo050035n

[49] Habuchi, T., Terao, Y. & Utsugi, M. A robust method using reducing inorganic salts for preventing the desulfurization of phosphorothioate oligonucleotides during the cleavage and deprotection step. *Tetrahedron Lett. 133*. (2023).

[50] Lietard, J. et al. Chemical and photochemical error rates in light-directed synthesis of complex DNA libraries. *Nucleic Acids Res.* **49** (12), 6687–6701 (2021). 34157124 10.1093/nar/gkab505 PMC8266620

[51] Lyng, H. et al. Profound influence of microarray scanner characteristics on gene expression ratios: analysis and procedure for correction. *BMC Genom.* **5** (1), 10 (2004). 10.1186/1471-2164-5-10 PMC356910 15018648

[52] Kibler-Herzog, L., Zon, G., Uznanski, B., Whittier, G. & Wilson, W. D. Duplex stabilities of phosphorothioate, methylphosphonate, and RNA analogs of two DNA 14-mers. *Nucleic Acids Res.* **19** (11), 2979–2986 (1991). 1711677 10.1093/nar/19.11.2979 PMC328260

[53] Hashem, G. M., Pham, L., Vaughan, M. R. & Gray, D. M. Hybrid Oligomer Duplexes Formed with Phosphorothioate DNAs: CD Spectra and Melting Temperatures of S-DNA·RNA Hybrids Are Sequence-Dependent but Consistent with Similar Heteronomous Conformations. *Biochemistry* **37** (1), 61–72 (1998). 9425026 10.1021/bi9713557

[54] Naiser, T. et al. Impact of point-mutations on the hybridization affinity of surface-bound DNA/DNA and RNA/DNA oligonucleotide-duplexes: comparison of single base mismatches and base bulges. *Bmc Biotechnol.* **8** (1), 48 (2008). 18477387 10.1186/1472-6750-8-48 PMC2435543

