# Supplementary material for: Synthesis of highly complex phosphorothioate-modified oligonucleotides on microarrays
Source: Sci Rep. 2026 Apr 28;16:18514. doi: 10.1038/s41598-026-50718-5 (PMC13269824; doi:10.1038/s41598-026-50718-5)
Supplement: Supplementary file 1 — Supplementary Material 1 [file 41598_2026_50718_MOESM1_ESM.pdf]

# SUPPORTING INFORMATION

**Synthesis of highly complex phosphorothioate-modified oligonucleotides on microarrays**

Igor Ilić[1,†], Erika Schaudy[1,†] and Jory Lietard[1,*]

[1] Institute of Inorganic Chemistry, Faculty of Chemistry, University of Vienna, Josef-Holaubek-Platz 2, 1090 Vienna, Austria

| Code | Array Sequence (5'-3') | Chemistry | Library size | Spot size | Replicates per array | Complement (5'-3') | Chemistry |
|---|---|---|---|---|---|---|---|
| 13mer | TCATGACGGTTAG | PS | $2^{12}$ (4 096) | 2 × 2 | 5-6 | CUAACCGUCAUGA | RNA |
| 15mer | AGTCCTGACATCGTG | PS | $2^{14}$ (16 384) | 1 × 1 | 11-12 | CACGAUGUCAGGACU | RNA |
| 18mer | ATATCCTTGTCGTATCCC | PS | $2^{17}$ (131 072) | 1 × 1 | 2-3 | GGGAUACGAUAAGGAUAU | RNA |

*Table S1.* Sequences used in this study. PS = phosphorothioate. Feature size corresponds to spot size on the microarray, 1 × 1 is a spot size using a single mirror of the DMD (14 × 14 $\mu m^2$), 2 × 2 is a square-shaped feature composed of 4 single-mirror spots. All complementary strands are labelled at the 5' end with a Cy3 dye.

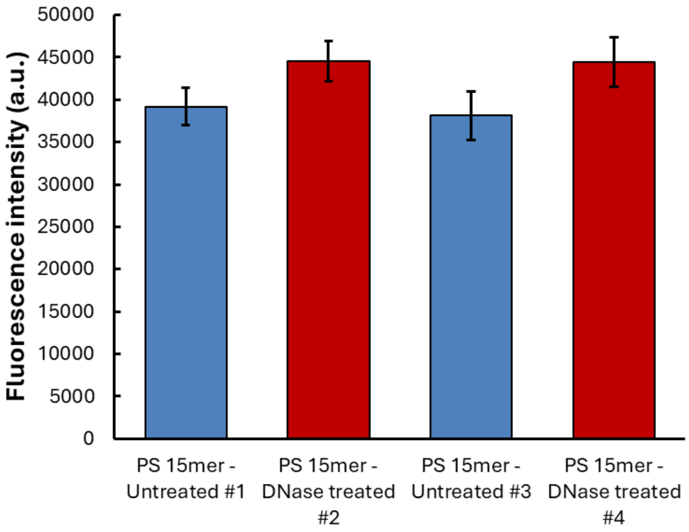


Figure S1. Hybridization assay performed on a microarray populated with a 15mer DNA sequence synthesized in four separate parts on the array (subarrays labeled #1 – 4). Synthesis was carried out with sulfurization at each step. After deprotection, subarrays #2 and #4 were treated with DNase I (0.02 and 0.04 u/µL, respectively, in 50 µL total reaction volume) for 10 min at 37°C. Subarrays #1 and #3 were left untreated. Each subarray was then hybridization to a Cy3-labeled complementary DNA strand. Error bars are SD.

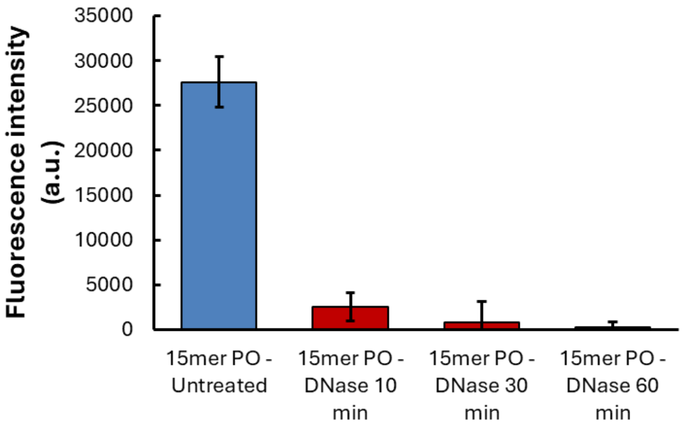


Figure S2. Hybridization assay performed on a microarray populated with a 15mer DNA sequence synthesized in four separate parts on the array. Synthesis was carried out with oxidation at each step (PO 15mer). After deprotection, subarrays #2-4 were treated with DNase I (0.02 u/µL, for 10, 30 or 60 min at 37°C). Subarray #1 was left untreated. Each subarray was then hybridized to a Cy3-labeled complementary DNA strand. Error bars are SD.

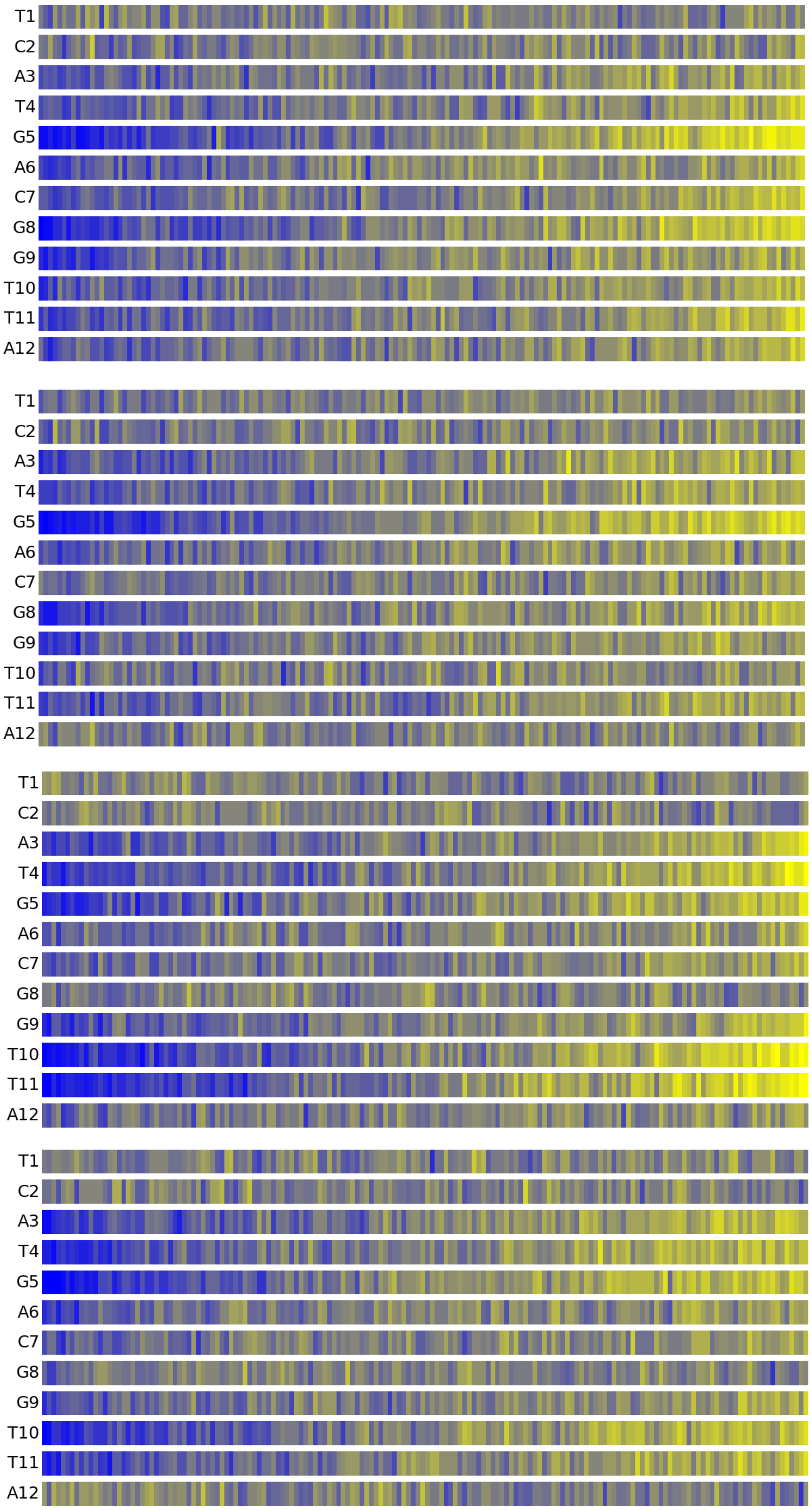
T1
C2
A3
T4
G5
A6
C7
G8
G9
T10
T11
A12
T1
C2
A3
T4
G5
A6
C7
G8
G9
T10
T11
A12
T1
C2
A3
T4
G5
A6
C7
G8
G9
T10
T11
A12
T1
C2
A3
T4
G5
A6
C7
G8
G9
T10
T11
A12

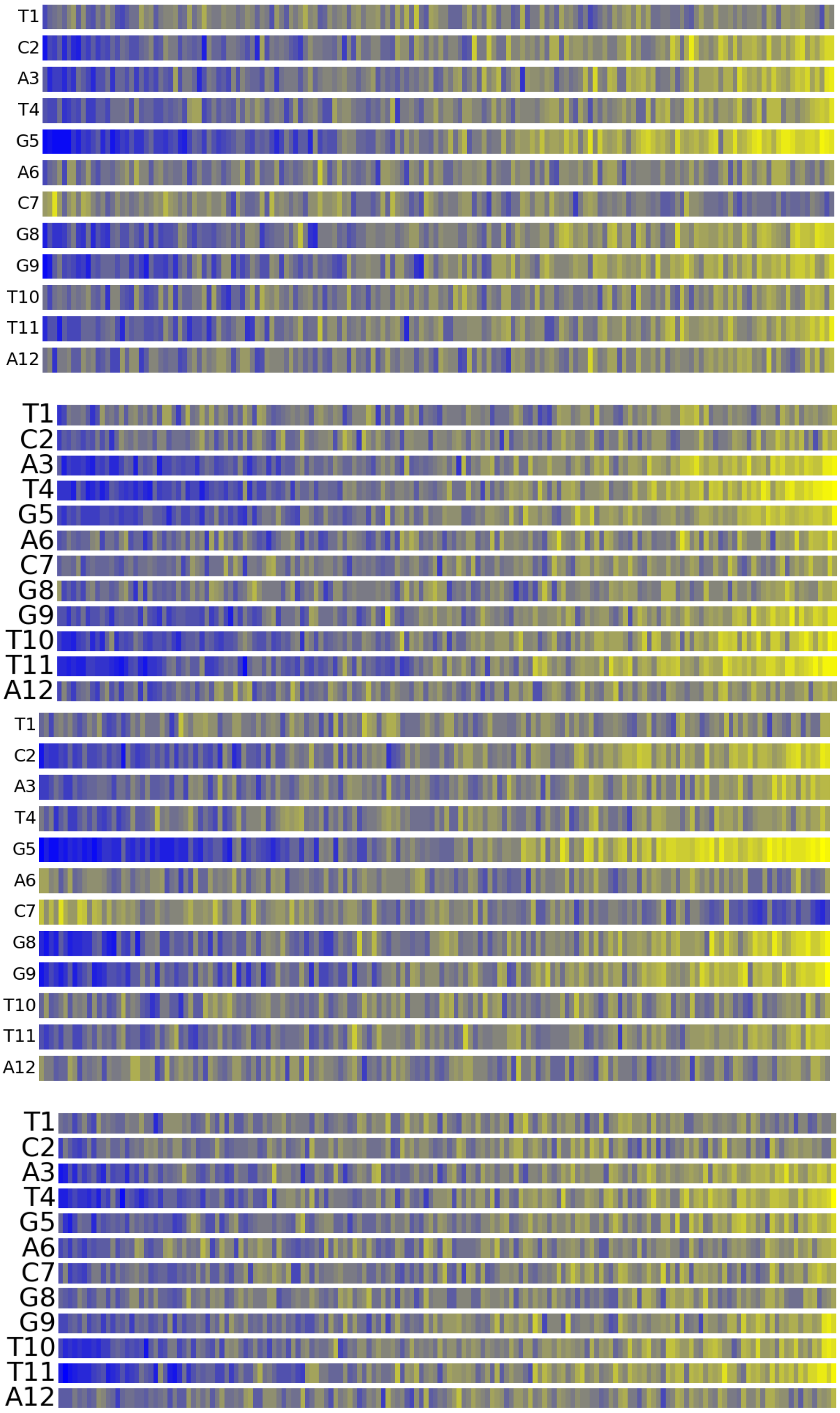


Figure S3. PO/PS distribution per linkage and as a function of hybridization signal for the 13mer, for each individual array. Binned distribution of fluorescence (bin size = 25 sequences for 13mer) arranged by internucleotidic linkage. For each bin at each linkage, a colour code was assigned corresponding to the relative frequency of PO or PS at that position. Bins are arranged from high to low fluorescence, left to right. In this raw dataset, "T1" indicates the nature of the phosphorus atom between T1 and C2, and "A12" the nature of the phosphorus atom between A12 and G13.

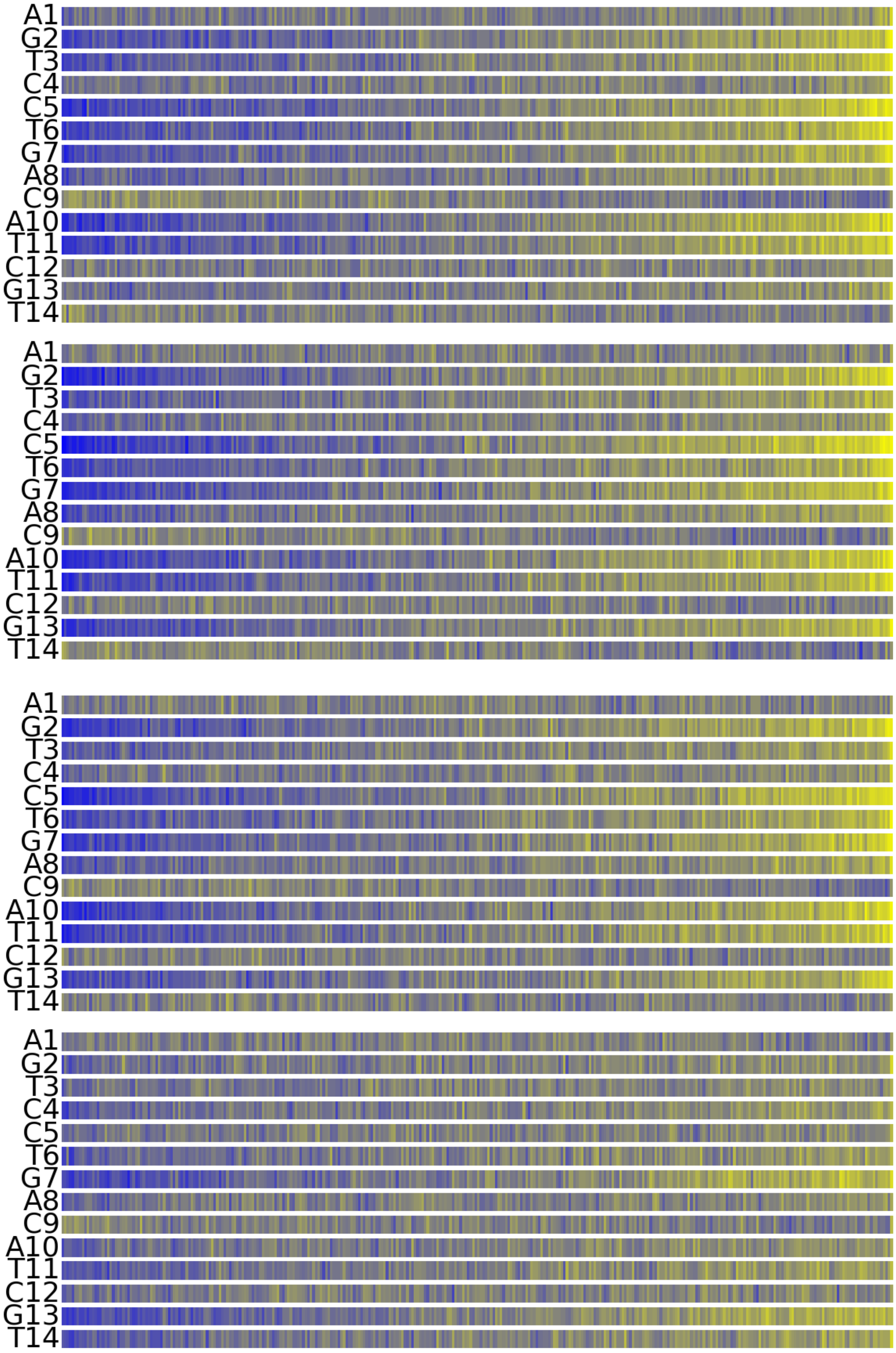
A1
G2
T3
C4
C5
T6
G7
A8
C9
A10
T11
C12
G13
T14

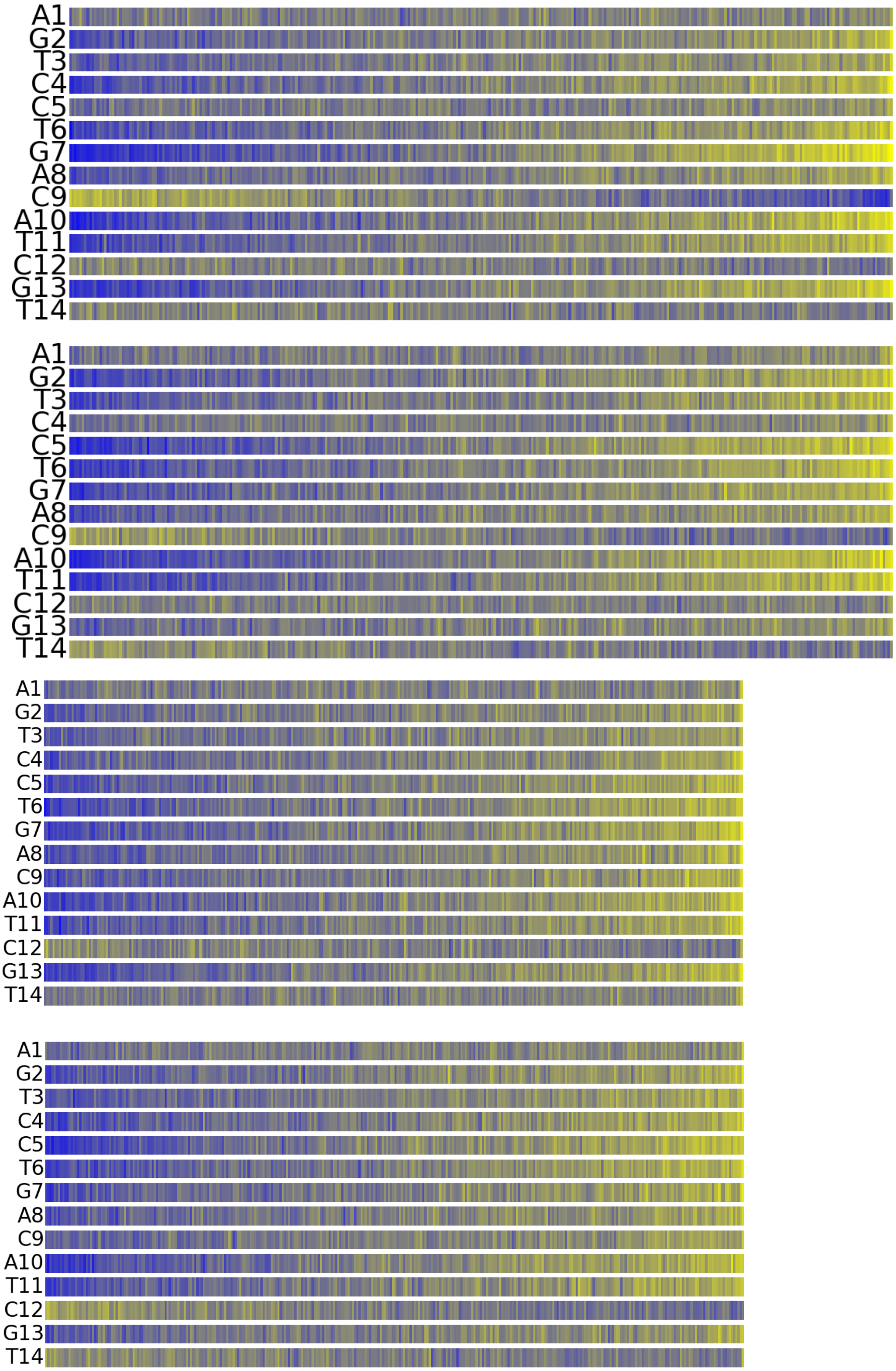
A1
G2
T3
C4
C5
T6
G7
A8
C9
A10
T11
C12
G13
T14
A1
G2
T3
C4
C5
T6
G7
A8
C9
A10
T11
C12
G13
T14
A1
G2
T3
C4
C5
T6
G7
A8
C9
A10
T11
C12
G13
T14
A1
G2
T3
C4
C5
T6
G7
A8
C9
A10
T11
C12
G13
T14

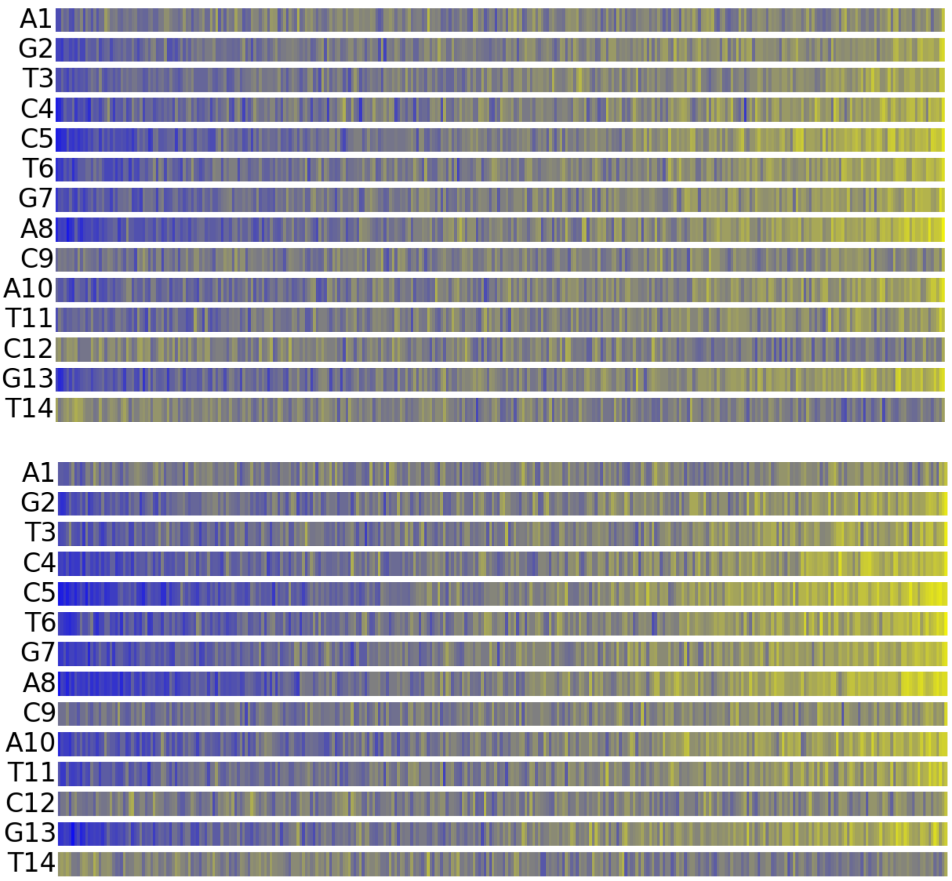


Figure S4. PO/PS distribution per linkage and as a function of hybridization signal for the 15mer, for each individual array. Binned distribution of fluorescence (bin size = 50 sequences) arranged by internucleotidic linkage. For each bin at each linkage, a colour code was assigned corresponding to the relative frequency of PO or PS at that position. Bins are arranged from high to low fluorescence, left to right. In this raw dataset, “A1” indicates the nature of the phosphorus atom between A1 and G2, and “T14” the nature of the phosphorus atom between T14 and G15.

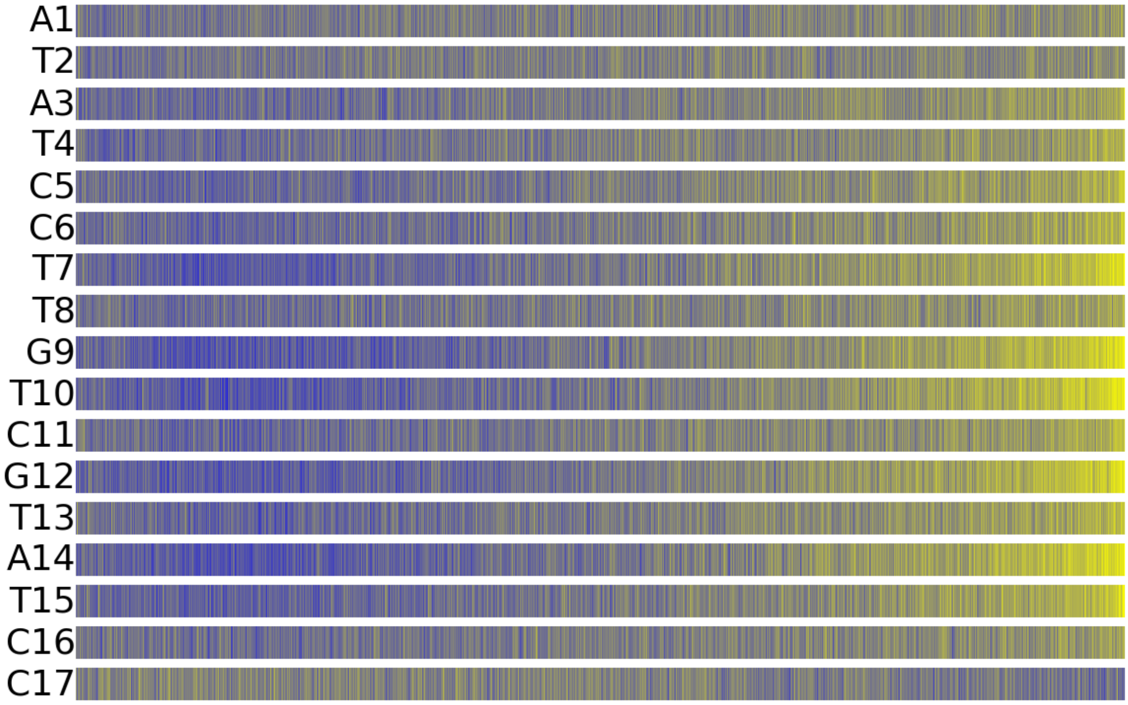
A1
T2
A3
T4
C5
C6
T7
T8
G9
T10
C11
G12
T13
A14
T15
C16
C17

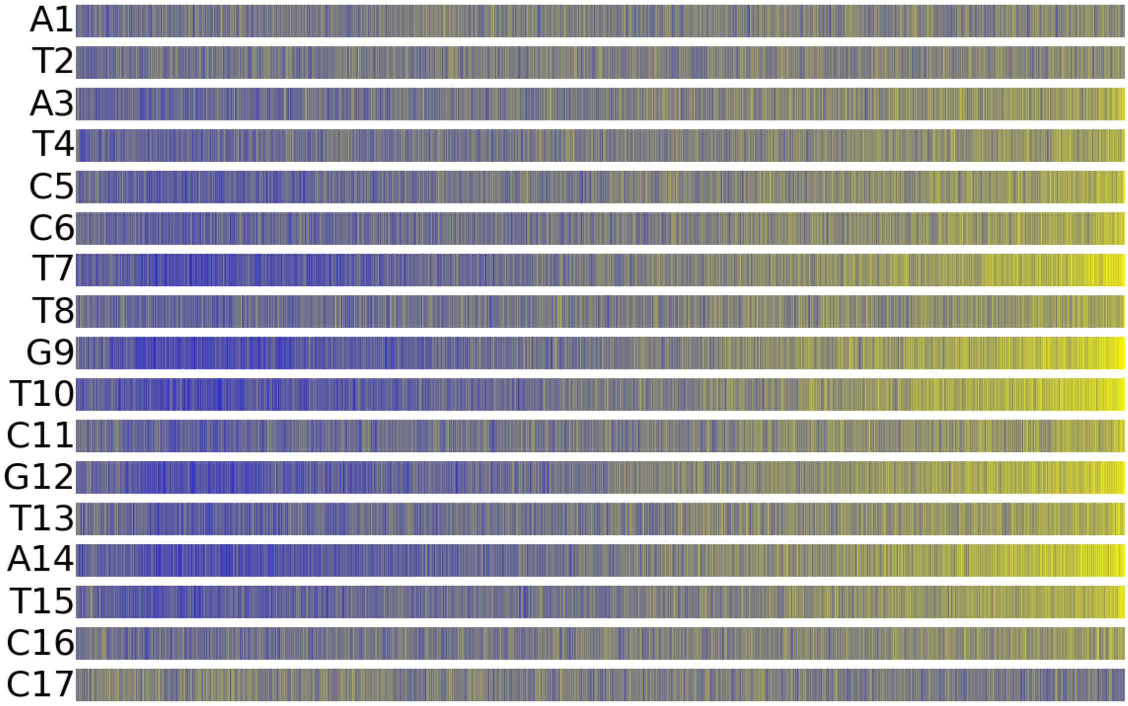
A1
T2
A3
T4
C5
C6
T7
T8
G9
T10
C11
G12
T13
A14
T15
C16
C17

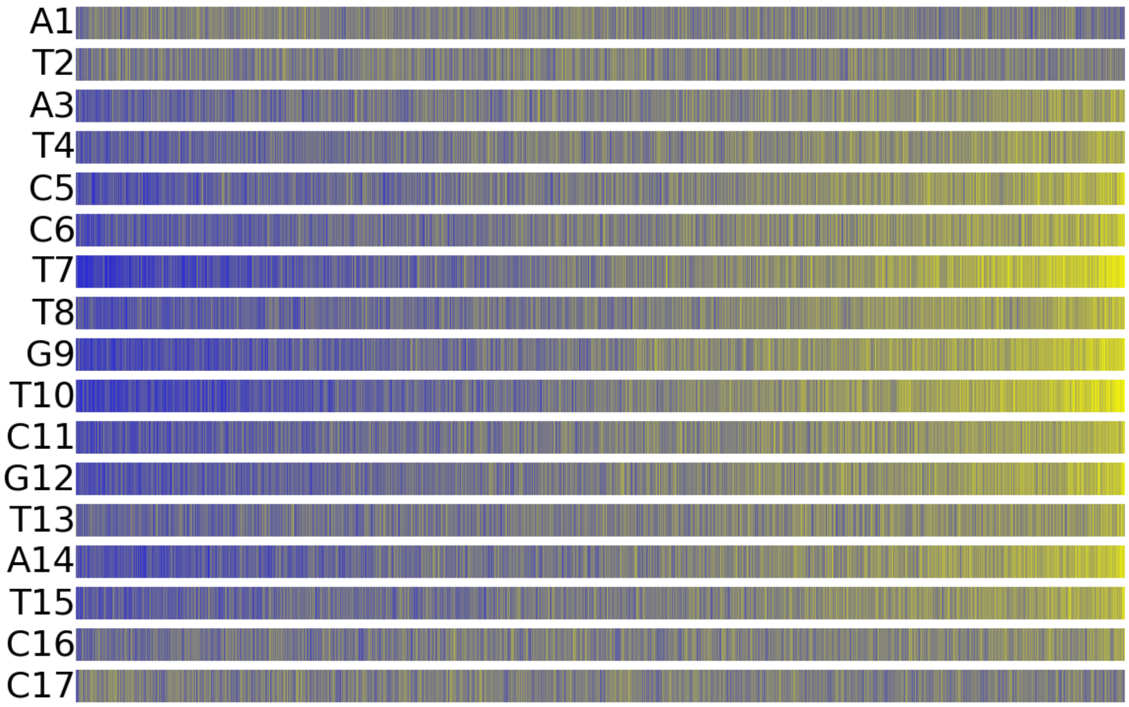
A1
T2
A3
T4
C5
C6
T7
T8
G9
T10
C11
G12
T13
A14
T15
C16
C17

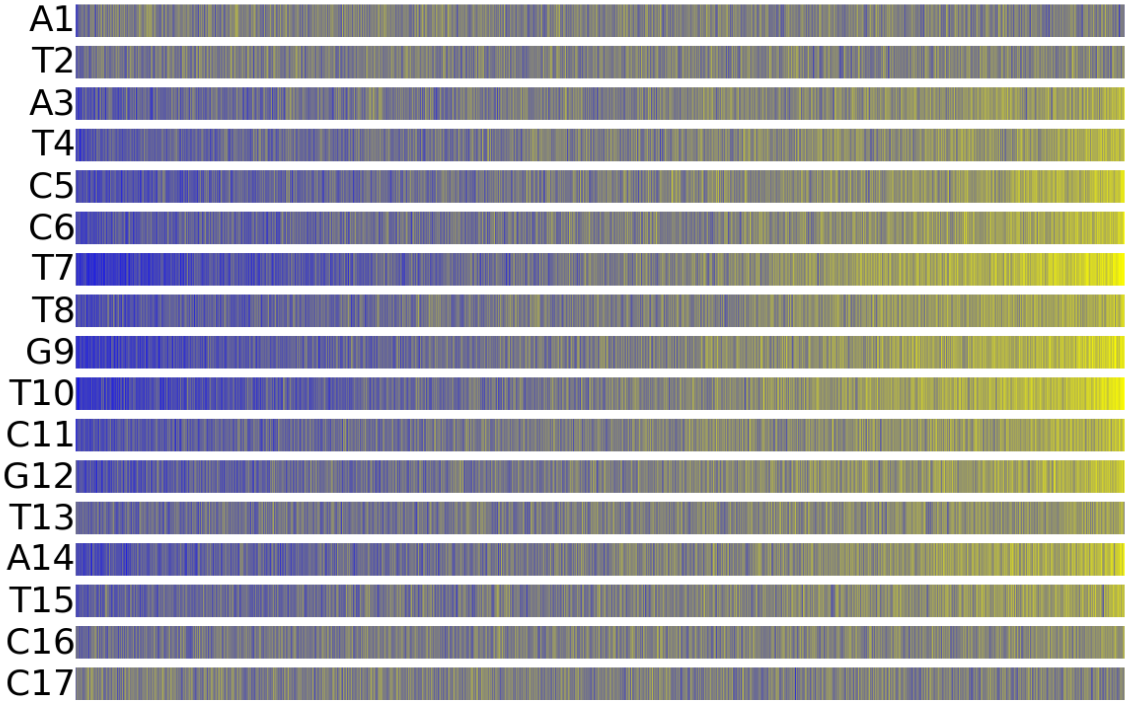
A1
T2
A3
T4
C5
C6
T7
T8
G9
T10
C11
G12
T13
A14
T15
C16
C17

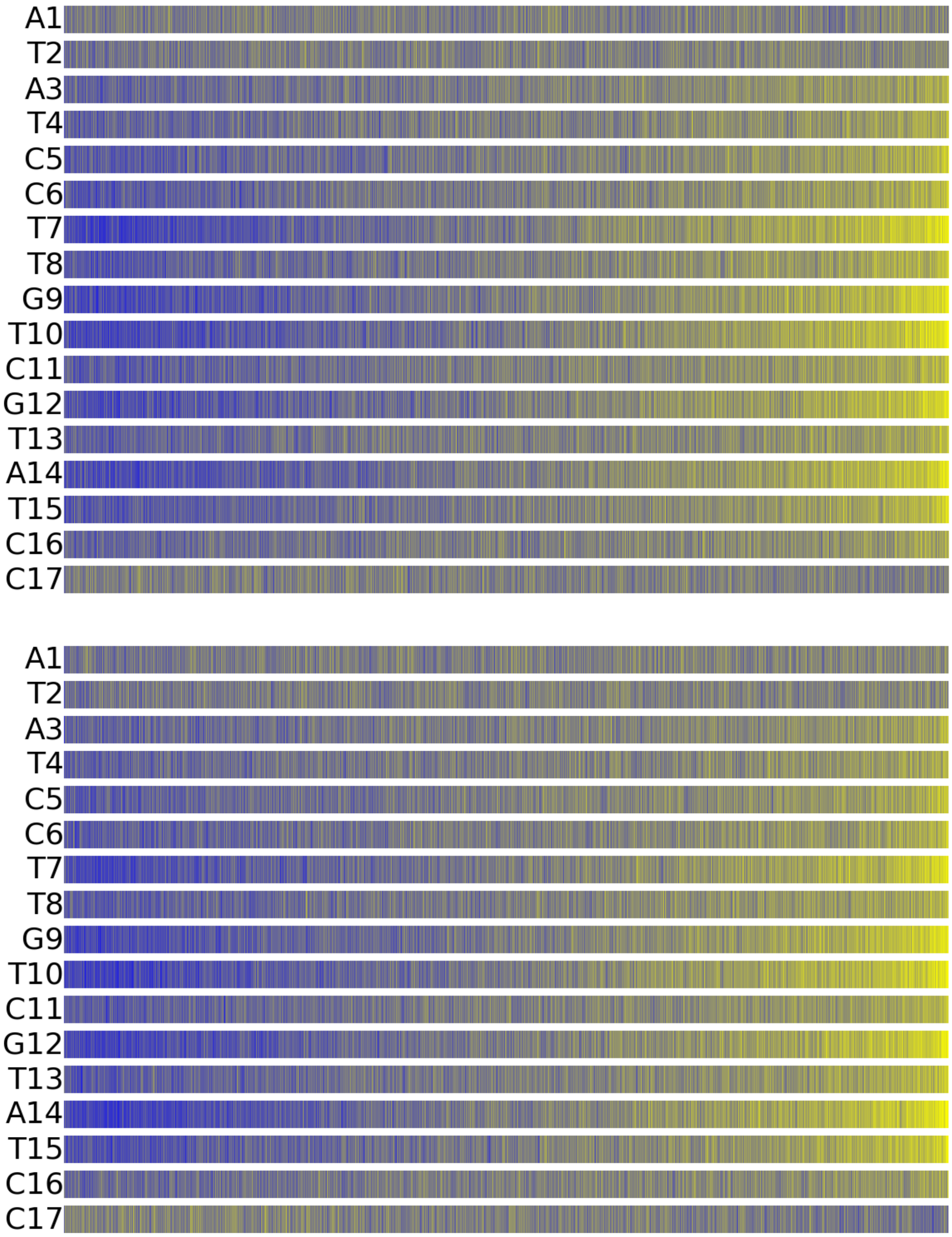


Figure S5. PO/PS distribution per linkage and as a function of hybridization signal for the 18mer, for each individual array. Binned distribution of fluorescence (bin size = 50 sequences) arranged by internucleotidic linkage. For each bin at each linkage, a colour code was assigned corresponding to the relative frequency of PO or PS at that position. Bins are arranged from high to low fluorescence, left to right. In this raw dataset, "A1" indicates the nature of the phosphorus atom between A1 and T2, and "C17" the nature of the phosphorus atom between C17 and C18.

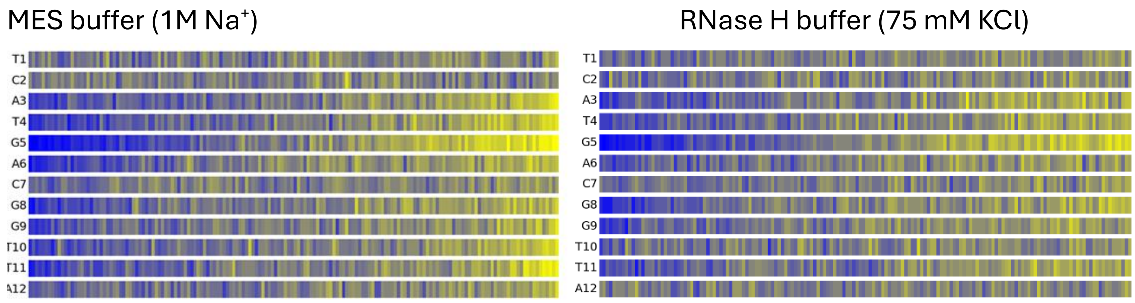


Figure S6. Comparison of binned distribution of fluorescence signal for the 13mer PO/PS library hybridized under high salt (MES buffer: 100 mM 2-morpholinoethanesulfonic acid, 1 M $Na^+$, 20 mM EDTA, and 0.01% Tween 20, left) or low salt conditions (RNase H buffer: 50 mM Tris-HCl, 75 mM KCl, 3 mM $MgCl_2$, 10 mM DTT, pH 8.3, right).

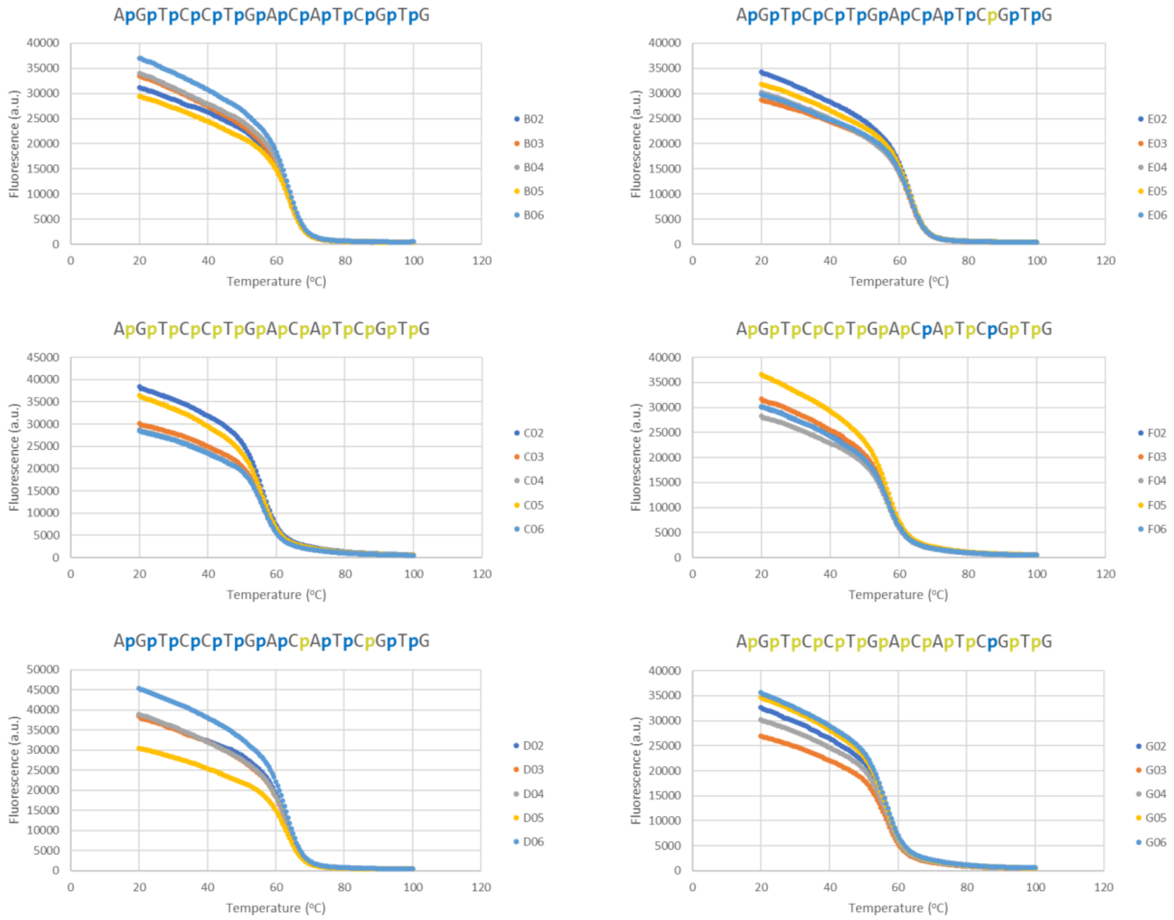

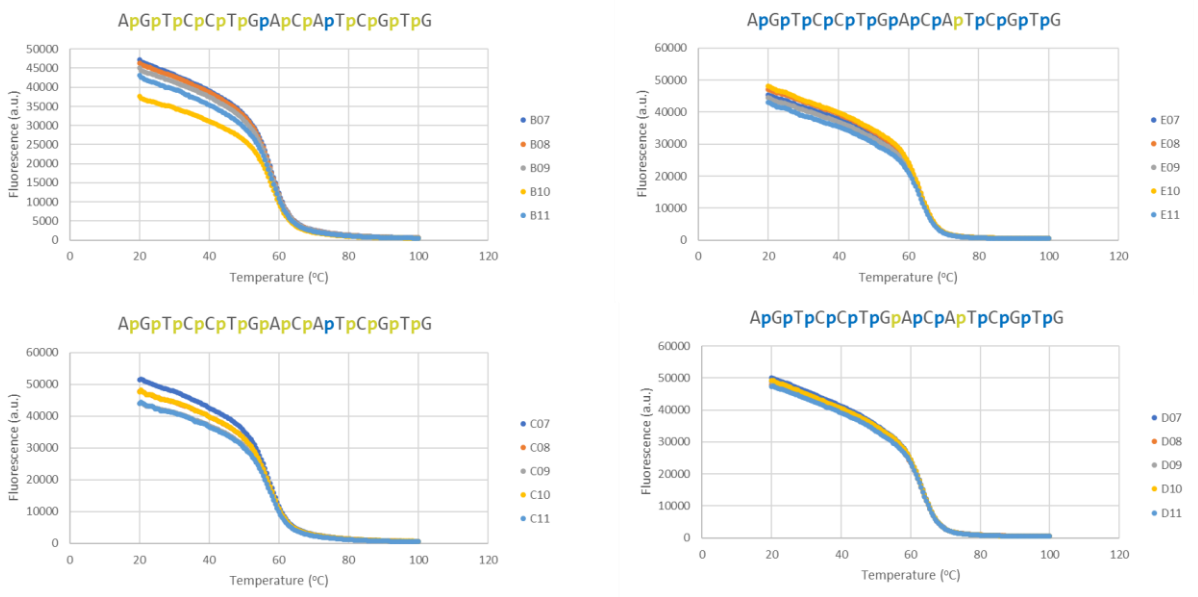


Figure S7. Representative fluorescence curves for high-resolution melting measurements. Each curves represents a separate well on the 96-well plate loaded onto the qPCR device. Each oligo (PO bond: blue, PS bond: yellow) was mixed in stoichiometric amount with its RNA complement in PBS buffer supplemented with EvaGreen dye. Curves correspond to heating cycles (20 to 100 °C) with decreasing fluorescence units (arbitrary units) as a function of temperature, corresponding to gradual duplex melting and release of the intercalating fluorescent dye. Tm were calculated using the first derivative of the sigmoidal transition.
